# Supplementary material for: Addressing the Knowledge Deficit in Hospital Bed Planning and Defining an Optimum Region for the Number of Different Types of Hospital Beds in an Effective Health Care System
Source: Int J Environ Res Public Health. 2023 Dec 12;20(24):7171. doi: 10.3390/ijerph20247171 (PMC11080941; doi:10.3390/ijerph20247171)
Supplement: Supplementary file 1 [file ijerph-20-07171-s001.zip › ijerph-2689026-supplementary.pdf]

**Supplementary Material S1.** Relevant publications by the author

The following is a list of publications arising from 30-years of research into the areas of forecasting healthcare demand, hospital bed modelling and the associated areas of healthcare costs and cost fluctuations (financial risk). All of which are interlinked. An extensive series of reports and discussion papers are also available via ResearchGate.

The papers are written in a non-academic style so that busy healthcare managers and policy makers can understand the issues. The fundamental insight comes from hard-won understanding by long experience and extensive supporting literature research. To avoid self-citation, articles will be referred to as A.1., B.11., etc., in the text. If you are unable to locate a full-text version of any paper, contact the author at hcaf_rod@yahoo.co.uk
BJHCM = British Journal of Healthcare Management. BJMMR is now J Adv Med Medical Res (JAMMR), articles archived at https://www.journaljammr.com/index.php/JAMMR/issue/archive.

**A. Understanding Emergency Admissions & Unscheduled Care**

1. Jones, R. 1997. Emergency admissions: Admissions of difficulty ***Health Service Journal*** 107(5546), 28-31.
2. Jones, R. 2009. Trends in emergency admissions. ***BJHCM*** 15(4), 188-196.
3. Jones, R. 2009. Cycles in emergency admissions. ***BJHCM*** 15(5), 239-246.
4. Jones, R. 2010. Emergency preparedness. ***BJHCM*** 16(2), 94-95.
5. Jones, R. 2010. Gender ratio and hospital admissions. ***BJHCM*** 16(11), 541.
6. Jones, R. 2011. Cycles in gender-related costs for long-term conditions. ***BJHCM*** 17(3), 124-125.
7. Jones, R. 2012. Gender ratio and cycles in population health costs. ***BJHCM*** 18(3), 164-165.
8. Jones, R. 2012. Environment induced volatility and cycles in population health. Positive Health Online 194 (May), http://www.positivehealth.com/article/clinicalpractice/environment-induced-volatility-and-cycles-in-population-health
9. Jones, R. 2013. Is the demographic shift the real problem? ***BJHCM*** 19(10), 509-511.
10. Jones, R. 2013. Trends in elderly diagnoses: links with multi-morbidity. ***BJHCM*** 19(11), 553-558.
11. Jones, R. 2014. What is happening in unscheduled care? ***J Paramed Pract*** 5(2), 60-62.
12. Jones, R. 2014. Forecasting conundrum: a disease time cascade. ***BJHCM*** 20(2), 90-91.
13. Jones, R. 2014. Long-term cycles in admissions for neurological conditions. ***BJHCM*** 20(4), 192-193.
14. Jones, R. 2014. Trends in admission for allergy. ***BJHCM*** 20(7), 350-351.
15. Jones, R. 2015. Forecasting medical emergency admissions. ***BJHCM*** 21(2), 98-99.
16. Jones, R. 2015. Estimating acute costs. ***BJHCM*** 21(3), 152-153.
17. Jones, R. 2015. Understanding growth in emergency admissions. ***BJHCM*** 21(4), 195-197.
18. Jones, R. 2015. Exploring trends in demand for urgent care. ***J Paramed Pract*** 7(10), 486-488.
19. Jones, R. 2016. The unprecedented growth in medical admissions in the UK: the ageing population or a possible infectious/immune aetiology? ***Epidemiology: Open Access*** 6(1), 1000219. The Unprecedented Growth in Medical Admissions in the UK: The Ageing Population or a Possible Infectious/Immune Aetiology? (omicsonline.org)
20. Jones, R. 2016. Rising emergency admissions in the UK and the elephant in the room. ***Epidemiology: Open Access*** 6(4), 1000261 Rising Emergency Admissions in the UK and the Elephant in the Room (omicsonline.org)

**B. Trends in Emergency Department Attendances & Subsequent Admission**

1. Jones, R. 2010. Forecasting emergency department attendances. ***BJHCM*** 16(10), 495-496.
2. Jones, R. 2012. Ambulance callouts and disruptive technology. ***BJHCM*** 18(2), 112-113.
3. Jones, R. 2012. Age-related changes in A&E attendance. ***BJHCM*** 18(9), 502-503.
4. Jones, R. 2013. Trends in unscheduled care. ***BJHCM*** 19(6), 301-304.
5. Jones, R. 2013. Hidden complexity in A&E trends in England. ***BJHCM*** 19(7), 354-355.
6. Jones, R. 2013. A&E attendance: the tip of a wider trend. ***BJHCM*** 19(9), 458-459.
7. Jones, R. 2014. Untangling the A&E crisis. ***BJHCM*** 20(5), 246-247.
8. Jones, R. 2015. A&E tipping points. ***BJHCM*** 21(6), 248-249.
9. Jones, R. 2015. A&E admissions: where next? ***BJHCM*** 21(6), 292.
10. Beeknoo N, Jones, R. 2016. Factors influencing A&E attendance, admissions and waiting times at two London hospitals. ***JAMMR*** 17(10), 1-29. View of Factors Influencing A & E Attendance, Admissions and Waiting Times at Two London Hospitals (journaljammr.com)
11. Beeknoo N, Jones, R. 2016. Using Social Groups to Locate Areas with High Emergency Department Attendance, Subsequent Inpatient Admission and Need for Critical Care. ***JAMMR*** 18(6), 1-23. View of Using Social Groups to Locate Areas with High Emergency Department Attendance, Subsequent Inpatient Admission and Need for Critical Care (journaljammr.com)

**C. Forecasting & Understanding Demand**

1. Jones, R. 1996. Estimation of annual activity and the use of activity multipliers. ***Health Informatics*** 2, 71-77.
2. Jones, R. 1996. How many patients next year? Healthcare Analysis & Forecasting, Camberley, UK.

3. Jones, R. 2010. Forecasting year-end activity. ***BJHCM*** 16(7), 350-351.
4. Jones, R. 2010. Forecasting demand. ***BJHCM*** 16(8), 392-393.
5. Jones, R. 2011. Cycles in inpatient waiting time. ***BJHCM*** 17(2), 80-81.
6. Jones, R. 2011. Death and future healthcare expenditure. ***BJHCM*** 17(9), 436-437.
7. Jones, R. 2012. Weathering the storm: Birth forecasting in turbulent times. ***Midwives Magazine*** *15*(2); https://www.rcm.org.uk/news-views-and-analysis/analysis/weathering-the-storm
8. Jones, R. 2014. Expected trends in births and deaths to 2037. ***BJHCM*** 20(8), 402-403.
9. Jones, R. 2015. Unexplained infectious events leading to deaths and medical admissions. ***BJHCM*** 21(1), 46-47.
10. Jones, R. 2015. Forecasting medical emergency admissions. ***BJHCM*** 21(2), 98-99.
11. Jones, R. 2015. Estimating acute costs. ***BJHCM*** 21(3), 152-153.
12. Jones, R. 2015. Understanding growth in emergency admissions. ***BJHCM*** 21(4), 195-197.
13. Jones, R. 2015. Trends in demand for urgent care. ***J Paramed Pract*** 7(10), 486-488.
14. Beeknoo N, Jones, R. 2016. Using social groups to locate areas of high utilization of critical care. ***BJHCM*** 22(11), 551-560.
15. Beeknoo N, Jones, R. 2017. The demography myth - how demographic forecasting vastly underestimates hospital admissions, and creates the illusion that fewer hospital beds or community-based bed equivalents will be required in the future. ***JAMMR 19(2), 1-27.*** View of The Demography Myth: How Demographic Forecasting Underestimates Hospital Admissions, and Creates the Illusion that Fewer Hospital Beds and Community-based bed Equivalents, will be Required in the Future (journaljammr.com)
16. Beeknoo, N.; Jones, R. 2017. Information asymmetry in financial forecasting within healthcare and simple methods to overcome this deficiency. ***JAMMR*** 20(4), 1-12. View of Information Asymmetry in Financial Forecasting within Healthcare and Simple Methods to Overcome this Deficiency (journaljammr.com)
17. Jones, R. 2017. What is driving growth in the English NHS? ***BJHCM*** 23(3), 134-137.
18. Jones, R. 2017. Volatility in emergency admissions per death. ***BJHCM*** 23(11), 552-554.
19. Jones, R. 2019. The nearness to death effect and why NHS pressures are going to intensify. ***J Paramed Pract*** 11(1), 28-30. https://www.magonlinelibrary.com/doi/10.12968/jpar.2019.11.1.28
20. Jones, R. 2019. Ignorance isn't bliss: behind the unequal distribution of end-of-life demand. ***J Paramed Pract*** 11(2), 77-79. https://www.magonlinelibrary.com/doi/abs/10.12968/jpar.2019.11.2.77
21. Jones, R. 2019. End-of-life demand is both highly volatile and shows unexpected trends. ***J Paramed Pract*** 11(3), 122-124. doi: 10.12968/jpar.2019.11.3.122
22. Jones, R. 2019. Unexplained periods of higher deaths contribute to marginal changes in health care demand and health insurance costs: International perspectives. ***International J Health Planning Management 35(3), 673-684.*** https://doi.org/10.1002/hpm.2917


**D. Trends in Outpatient Attendance and Follow-up to First Appointment ratio**


1. Beauchant, S.; Jones, R. 1997. Socio-economic and demographic factors in patient non-attendance. ***BJHCM*** 3(10), 523-528.
2. Jones, R. 2000) Outpatient appointments: Feeling a bit peaky. ***Health Service Journal*** 110(5732), 28-31.
3. Jones, R. 2001) Outpatient appointments: A pretty little sum. ***Health Service Journal*** 111(5740), 28-31.
4. Jones, R. 2001) Outpatient waiting times: Quick, quick, slow. ***Health Service Journal*** 111(5778), 20-23.
5. Jones, R. 2009. What next for 18 weeks? ***BJHCM*** 15(8), 404-405.
6. Jones, R. 2009. How to maintain 18 weeks. ***BJHCM*** 15(9), 456-457.
7. Jones, R. 2012. Are there cycles in outpatient costs? ***BJHCM*** 18(5), 276-277.
8. Jones, R. 2012. Increasing GP referrals: collective jump or infectious push? ***BJHCM*** 18(9), 487-495.
9. Jones, R. 2012. GP referral to dermatology: which conditions? ***BJHCM*** 18(11), 594-596.
10. Jones, R. 2012. Trends in outpatient follow-up rates, England 1987/88 to 2010/11. ***BJHCM*** 18(12), 647-655.
11. Jones, R. 2014. Unexpected changes in outpatient first attendance. ***BJHCM*** 20(3), 142-143.
12. Jones, R. 2016. Recent trends in outpatient follow-up rates. ***BJHCM*** 22(2), 92-94.


**E. Understanding Sickness Absence Rates – which follow the same curious patterns as deaths (see below)**


1. Jones, R. 2016. Unusual trends in NHS staff sickness absence. ***BJHCM*** 22(4), 239-240.
2. Jones, R. 2019. NHS sickness absence – the hidden message that no one is listening to. doi: 10.13140/RG.2.2.13996.31365
3. Jones, R. 2019. Sickness absence trends for the Department for Work & Pensions (England) follow identical hidden on/off patterns to those seen for NHS staff. doi: 10.13140/RG.2.2.27457
4. Jones, R. 2019. All-cause mortality and NHS sickness absence rates in England show a lagged series of step-like changes. ***Achievements of Biology and Medicine*** (Transl) 33(1), 41-43.
5. Jones, R. 2020. NHS sickness absence in England – hidden patterns. ***BJHCM*** 26(4), 1-11. http://doi.org.10.12968/bjhc.2019.0026

6. Jones, R. 2021. Multidisciplinary insights into health care financial risk and hospital surge capacity, Part 2: High population density is associated with enhanced year-to-year volatility in many aspects of poor health including health care worker sickness absence. ***Journal of Health Care Finance. 47(3),*** Multidisciplinary Insights into Health Care Financial Risk and Hospital Surge Capacity, Part 2: High Population Density is Associated with Enhanced Year-to-Year Volatility in Many Aspects of Poor Health Including Health Care Worker Sickness Absence | Jones, PhD | Journal of Health Care Finance (healthfinancejournal.com)

**F. Understanding Hospital Mortality**

1. Jones, R. 2015. A 'fatal' flaw in hospital mortality models: How spatiotemporal variation in all-cause mortality invalidates hidden assumptions in the models. ***FGNAMB*** 1(3), 82-96. doi: 10.15761/FGNAMB.1000116
2. Jones, R. 2015. Links between bed occupancy, deaths and costs. ***BJHCM*** 21(11), 544-545.
3. Jones, R. 2016. Hospital bed occupancy and deaths (all-cause mortality) in 2015. ***BJHCM*** 22(5), 283-285.
4. Jones, R. 2016. Clear the decks of Summary Hospital-level Mortality Indicator. ***BJHCM*** 22(6), 335-338.
5. Jones, R. 2016. Bed occupancy and hospital mortality. ***BJHCM*** 22(7), 380-381
6. Jones, R. 2016. Hospital deaths and length of stay. ***BJHCM*** 22(8), 424-425.
7. Jones, R. 2016. Hospital mortality rates and changes in activity. ***BJHCM*** 22(10), 519-521.
8. Jones, R.; Sleet G, Pearce O, Wetherill M 2016. Complex changes in blood biochemistry revealed by a composite score derived from Principal Component Analysis: Effects of age, patient acuity, end of life, day-of week, and potential insights into the issues surrounding the 'Weekend' effect in hospital mortality. ***JAMMR*** 18(5), 1-28. doi: 10.9734/BJMMR/2016/29355
9. Jones, R. 2016. Trends in proportion of deaths occurring in hospital. ***BJHCM*** 22 (11), 572-573.
10. Jones, R. 2016. Trends in crude death rates in English hospitals. ***BJHCM*** 22 (12), 616-617.
11. Jones, R. 2017. Is the 'weekend' mortality effect real? ***BJHCM*** 23 (1), 39-41.
12. Jones, R. 2017. In-hospital deaths, all-cause mortality and medical admissions. ***BJHCM*** 23(5), 239-240.
13. Jones, R. 2018. Hospital mortality scores are unduly influenced by changes in the number of admissions. ***European Journal of Internal Medicine*** 51: e35-e37. https://doi.org/10.1016/j.ejim.2018.02.010
14. Jones, R. 2018. Unexpected trends in hospital standardized mortality indicate a novel cause. ***Eur J Internal Med***. 52: e9-e11. https://doi.org/10.1016/j.ejim.2018.02.018
15. Jones, R. 2018. Hospital mortality scores are unduly influenced by changes in service configuration. ***BJHCM*** 24 (6), 297-301.

**G. Covid-19 and excess all-cause mortality**

1. Jones, R. 2020. How many extra deaths really occurred in the UK? See http://www.hcaf.biz/2020/Covid_Excess_Deaths.pdf
2. Jones, R. 2021. The COVID-19 counting fiasco: Is the real total of deaths closer to 10 million? In-depth analysis from India and other countries. ***Journal of Health Care Finance 47(3),*** Spring: Special Edition, Special Guest Authors (healthfinancejournal.com)
3. Jones, R. 2021. Did the government of India mislead the world regarding the extent of the COVID-19 problem in its constituent states? ***Journal of Health Care Finance 47(3),*** Spring Special Edition, Special Guest Authors (healthfinancejournal.com)
4. Jones, R. 2021. The true COVID-19 death toll in India is likely to be greater than 1.2 million persons. ***Journal of Health Care Finance 47(3),*** Spring Special Edition, Special Guest Authors (healthfinancejournal.com)
5. Jones, R. 2021. Low COVID-19 testing in the majority of nations has resulted in gross undercounting of infections and deaths. ***Journal of Health Care Finance 47(4),*** Fall Special Edition, Special Guest Authors (healthfinancejournal.com)
6. Jones, R.; Ponomarenko, A. 2023. COVID-related age profiles for SARS-CoV-2 variants in England and Wales and states of the USA (2020 to 2022): impact on all-cause mortality. Infectious Disease Reports 15,600-634.
7. Jones, R.; Ponomarenko, A. 2023. Effect of age, sex, and COVID-19 vaccination history on all-cause mortality: unexpected outcomes in a complex biological and social system. Effect of Age, Sex, and COVID-19 Vaccination History on All-Cause Mortality: Unexpected Outcomes in a Complex Biological and Social System [v1] | Preprints.org
8. Jones, R.; Ponomarenko, A. Pathogens have different age/sex profiles for hospital admission and why COVID-19 variants may behave as if they were 'different' pathogens. Pathogens Have Different Age/Sex Profiles for Hospital Admission and Why COVID-19 Variants Behave as If They Were 'Different' Pathogens[v1] | Preprints.org

**H. Understanding Excess Winter Mortality (EWM) and Winter Capacity Planning**

1. Jones, R. 2017. The link between seasonal death rates and workloads. ***BJHCM*** 23(9), 448-450.
2. Jones, R. 2017. Anticipated ambulance workload during the 2016/17 winter**.** ***J Paramed Pract*** 9(2), 52-54.
3. Jones, R. 2019. Does on/off switching of deaths modify NHS winter workload? ***J Paramed Pract*** 11(4), 172-173.
4. Jones, R. 2019. Will the winter of 2019/2020 have unusually high service demand? Part 1: Lessons. ***J Paramed Pract*** 11(11), 492-494.
5. Jones, R. 2019. Will the winter of 2019/2020 have unusually high service demand? Part 2: Strategy. ***J Paramed Pract*** 11(12), 538-540.
6. Jones, R. 2020. Excess winter mortality (EWM) and stalling international improvements in life expectancy and mortality rates. ***BJHCM*** 26(12.; https://doi.org/10.12968/bjhc.2020.0020
7. Jones, R. 2021. Excess winter mortality (EWM) as a dynamic forensic tool: Where, when, which conditions, gender, ethnicity, and age. ***Int J Environ Res Public Health*** 18(4); 2161. https://doi.org/10.3390/ijerph18042161
8. Jones, R.; Ponomarenko, A. 2022. Trends in excess winter mortality (EWM) from 1900/01 to 2019/20 – evidence for a complex system of multiple long-term trends. ***Int J Environ Res Public Health*** 19, 3407. https://doi.org/10.3390/ijerph19063407
9. Jones, R.; Ponomarenko, A. 2022. System complexity in influenza infection and vaccination: effects upon excess winter mortality. ***Infectious Disease Reports*** 14(3), 287-309. https://doi.org/10.3390/idr14030035
10. Jones, R. 2023. Could some vaccines have unanticipated effects against NHS winter pressures? ***J Paramed Pract*** 15(6), 6-9.
11. Jones, R. 2023. Winter pressures: policy versus reality. ***J Paramed Pract*** 15(7), 170-172.
12. Jones, R.; Ponomarenko, A 2023. COVID-19-Related Age Profiles for SARS-CoV-2 Variants in England and Wales and States of the USA (2020 to 2022): Impact on All-Cause Mortality. Infectious Disease Reports 15(5); 600-634. Infectious Disease Reports | Free Full-Text | COVID-19-Related Age Profiles for SARS-CoV-2 Variants in England and Wales and States of the USA (2020 to 2022): Impact on All-Cause Mortality (mdpi.com)

**I. Excess all-cause mortality in influenza and COVID-19 vaccination**

1. Jones, R.; Ponomarenko, A. 2022. Trends in excess winter mortality (EWM) from 1900/01 to 2019/20 – evidence for a complex system of multiple long-term trends. ***Int J Environ Res Public Health*** 19, 3407. https://doi.org/10.3390/ijerph19063407
2. Jones, R.; Ponomarenko, A. 2022. System complexity in influenza infection and vaccination: effects upon excess winter mortality. ***Infectious Disease Reports*** 14(3), 287-309. https://doi.org/10.3390/idr14030035
3. Jones, R.; Ponomarenko, A 2022. Roles for pathogen interference in influenza vaccination, with implications to vaccine effectiveness (VE) and attribution of influenza deaths. ***Infectious Disease Reports*** 14(5), 710- 758. https://doi.org/10.3390/idr14050076
4. Jones, R.; Ponomarenko, A. Roles for age, gender, vaccination history and COVID-19 variants in all-cause mortality: unexpected outcomes in a complex system. ***In review*** Effect of Age, Sex, and COVID-19 Vaccination History on All-Cause Mortality: Unexpected Outcomes in a Complex Biological and Social System [v1] | Preprints.org

**J. Was Austerity Directly Linked to Higher Deaths?**

1. Jones, R. 2017. Did austerity cause the rise in deaths seen in England and Wales in 2015? ***BJHCM*** 23(9), 418-424.
2. Jones, R. 2017. Essays on rising mortality in England and Wales – a MEDLINE search is not infallible**.** ***J Roy Soc Med (JRSM) 110(6),224.*** doi: 10.1177/0141076817703864
3. Jones, R. 2017. Role of social group and gender in outbreaks of a novel agent leading to increased deaths, with insights into higher international deaths in 2015. ***Fractal Geometry and Nonlinear Analysis in Medicine and Biology*** 3(1), 1-7. doi: 10.15761/FGNAMB.1000146
4. Jones, R. 2017. Different patterns of male and female deaths in 2015 in English and Welsh local authorities question the role of austerity as the primary force behind higher deaths. ***Fractal Geometry and Nonlinear Analysis in Medicine and Biology*** 3(1), 1-4. doi: 10.15761/FGNAMB.1000145
5. Jones, R. 2019. Unanswered questions for the austerity theory. Healthcare Analysis & Forecasting. doi: 10.13140/RG.2.2.20357.60643 and also https://papers.ssrn.com/sol3/papers.cfm?abstract_id=3319211
6. Jones, R. 2019. Deaths in England and Wales are falling – does the austerity theory still apply? Healthcare Analysis & Forecasting. Available via SSRN. See https://papers.ssrn.com/sol3/papers.cfm?abstract_id=3381403
7. Jones, R. 2019. Austerity in the UK and poor health: were deaths directly affected? ***BJHCM*** 25(11), 337-347.
8. Jones, R. 2020. Excess winter mortality (EWM) and stalling international improvements in life expectancy and mortality rates. ***BJHCM*** 26(12).; https://doi.org/10.12968/bjhc.2020.0020

**K. Understanding Hospital Length of Stay (LOS) – central to the next section on bed planning**

1. Jones, R. 2009. Length of stay efficiency. ***BJHCM*** 15(11), 563-564.
2. Jones, R. 2010. Benchmarking length of stay. ***BJHCM*** 16(5), 248-250.
3. Jones, R. 2013. Average length of stay in hospitals in the USA. ***BJHCM*** 19(4), 186-191.
4. Jones, R. 2015. Is length of stay a reliable efficiency measure? ***BJHCM*** 21(7), 344-345.
5. Jones, R. 2015. Declining length of stay and future bed numbers. ***BJHCM*** 21(9), 440-441.
6. Jones, R. 2016. Hospital deaths and length of stay. ***BJHCM*** 22(8), 424-425.
7. Jones, R. 2016. Where next for overnight stay admissions, length of stay and bed days? ***BJHCM*** 22(9), 475-477.
8. Jones, R. 2017. Growth in NHS admissions and length of stay: A policy-based evidence fiasco. ***BJHCM*** 23(12), 603-606.
9. Jones, R. 2018. Maternity length of stay efficiency and neonatal admissions. ***BJHCM*** 24(3), 122-124.

**L. Understanding Hospital Bed Planning & Occupancy - bed demand is intrinsically linked to the forecasting demand section.**

1. Jones, R. 1997. Emergency admissions: Admissions of difficulty ***Health Service Journal*** 107(5546), 28-31.
2. Jones, R. 2001. Bed occupancy: Don't take it lying down. Health Service Journal 111(5752), 28-31.
3. Jones, R. 2001. New approaches to bed utilisation – making queuing theory practical. Presented at New Techniques for Health and Social Care. Harrogate Management Centre Conference 27th September 2001.
4. Jones, R.. 2002. Principles for effective bed planning and management. Healthcare Analysis & Forecasting. (PDF) Principles for effective bed planning & management (researchgate.net)
5. Jones, R. 2003. Bed management - Tools to aid the correct allocation of hospital beds. Presented at ***Re-thinking bed management – Opportunities and challenges***. Harrogate Management Centre Conference, 27th January 2003. Presented at Re-thinking Bed Management Opportunities & Challenges. Harrogate Management Centre Conference, London, Thursday 27 th February, 2003 (1library.net)
6. Jones, R. 2009. Emergency admissions and hospital beds. ***BJHCM*** 15(6), 289-296.
7. Jones, R. 2009. Building smaller hospitals. ***BJHCM*** 15(10), 511-512.
8. Jones, R. 2009. Crafting efficient bed pools. ***BJHCM*** 15(12), 614-616.
9. Jones, R. 2010. Myths of ideal hospital size. ***Medical Journal of Australia*** 193(5), 298-300.
10. Jones, R. 2011. Does hospital bed demand depend more on death than demography? ***BJHCM*** 17(5), 190-197. https://doi.org/10.12968/bjhc.2011.17.5.190 1/Method_for_determining_hospital_beds.pdf
11. Jones, R. 2011. Bed days per death: a new performance measure. ***BJHCM*** 17(5), 213.
12. Jones, R. 2011. Hospital bed occupancy demystified. ***BJHCM*** 17(6), 242-248.
13. Jones, R. 2011. A&E performance and inpatient bed occupancy. ***BJHCM*** 17(6), 256-257.
14. Jones, R. 2011. Bed occupancy – the impact on hospital planning. ***BJHCM*** 17(7), 307-313.
15. Jones, R. 2011. The need for single room accommodation in hospital. ***BJHCM*** 17(7), 316-317.
16. Jones, R. 2011. Demand for hospital beds in English Primary Care Organisations. ***BJHCM*** 17(8), 360-367.
17. Jones, R. 2011. A paradigm shift for bed occupancy. ***BJHCM*** 17(8), 376-377.
18. Jones, R. 2011. Volatility in bed occupancy for emergency admissions. ***BJHCM*** 17(9), 424-430.
19. Jones, R. 2011. A report investigating bed capacity at the Royal North Shore Hospital, NSW, Australia. Available online: (PDF) A Report Investigating Bed Capacity at the Royal North Shore Hospital (researchgate.net)
20. Jones, R. 2012. Maternity bed occupancy: all part of the equation. **Midwives Magazine** 15(1), http://www.rcm.org.uk/midwives/features/all-part-of-the-equation/
21. Jones, R. 2012. A simple guide to a complex problem – maternity bed occupancy. ***British Journal of Midwifery*** 20(5), 351-357.
22. Jones, R. 2013. A guide to maternity costs – why smaller units have higher costs. ***British Journal of Midwifery*** 21(1), 54-59.
23. Jones, R. 2013. Optimum bed occupancy in psychiatric hospitals. ***Psychiatry On-Line*** http://www.priory.com/psychiatry/psychiatric_beds.htm
24. Jones, R. 2013. The NHS England review of urgent and emergency care. ***BJHCM*** 19(8), 406-407.
25. Jones, R. 2014. Medical bed occupancy and cancelled operations. ***BJHCM*** 20(12), 594-595.
26. Jones, R. 2015. A&E tipping points. ***BJHCM*** 21(5), 248-249.
27. Jones, R. 2015. Bed occupancy, efficiency and infectious outbreaks. ***BJHCM*** 21(8), 396-397.
28. Jones, R. 2015. Links between bed occupancy, deaths and costs. ***BJHCM*** 21(11), 544-545.
29. Jones, R. 2016. Hospital bed occupancy and deaths (all-cause mortality) in 2015. ***BJHCM*** 22(5), 283-285.
30. Beeknoo N, Jones, R. 2016. Achieving economy of scale in critical care, and planning information necessary to support the choice of bed numbers. ***JAMMR*** 17(9),1-15. Achieving Economy of Scale in Critical Care, Planning Information Necessary to Support the Choice of Bed Numbers | *JAMMR* (journaljammr.com)

31. Beeknoo N, Jones, R. 2016. A simple method to forecast next years bed requirements: a pragmatic alternative to queuing theory. ***JAMMR*** 18(4), 1-20. A Simple Method to Forecast Future Bed Requirements: A Pragmatic Alternative to Queuing Theory | *JAMMR* (journaljammr.com)
32. Beeknoo, N.; Jones, R. 2016. The demography myth - how demographic forecasting underestimates hospital admissions and creates the illusion that fewer hospital beds or community-based bed equivalents will be required in the future. ***JAMMR* 19(2), 1-27.** The Demography Myth: How Demographic Forecasting Underestimates Hospital Admissions, and Creates the Illusion that Fewer Hospital Beds and Community-based bed Equivalents, will be Required in the Future | *JAMMR* (journaljammr.com)
33. Jones, R. 2016. Bed occupancy and hospital mortality. ***BJHCM*** 22(7), 380-381.
34. Jones, R. 2017. Is there scope to close acute beds in the STPs. ***BJHCM*** 23(2), 83-85.
35. Jones, R. 2017. What is driving growth in the English NHS? ***BJHCM*** 23(3), 134-137.
36. Jones, R. 2017. Flexibility, hospital bed numbers, and sustainability and transformation plans. ***BJHCM*** 23(7),344-345.
37. Jones, R. 2017. Deaths and acute hospital beds in the Sustainability and Transformation Plans. ***BJHCM*** 23(10), 498-499.
38. Jones, R. 2017. Bed occupancy continues to show on/off switching. ***BJHCM*** 23(11), 515-516.
39. Jones, R. 2018. Local 7-day patterns of on/off switching in acute bed occupancy. ***BJHCM*** 24(2), 100-102.
40. Jones, R. 2018. Do outbreaks of 'Disease X' regulate NHS beds and costs? ***BJHCM*** 24(4), 204-205.
41. Jones, R. 2018. Trends in available beds per death in England. ***BJHCM*** 24(7), 358-359.
42. Jones, R. 2018. Trends in critical care bed numbers in England. ***BJHCM*** 24(10), 516-517.
43. Jones, R. 2018. Hospital beds per death how does the UK compare globally. ***BJHCM*** 24(12), 617-622.
44. Jones, R. 2019. Condition specific growth in occupied beds in England following a sudden and unexpected increase in deaths. ***BJHCM*** 25(6), 1-8.
45. Jones, R. 2019. Have doctors and the public been misled regarding hospital bed requirements? ***BJHCM*** 25 (7), 242-250.
46. Jones, R. 2019. A pragmatic method to compare hospital bed provision between countries and regions: Beds in the states of Australia. ***Intl J Health Plan Mgmt*** 35(3), 746-759. https://doi.org/10.1002/hpm.2950
47. Jones, R. 2020. Curious patterns in hospital bed occupancy data. ***BJHCM*** 25(3), 71-72. https://doi.org/10.12968/bjhc.2020.0014
48. Jones, R. 2020. Would the United States have had too few beds for universal emergency care in the event of a more widespread Covid-19 epidemic? ***Int J Environmental Research and Public Health*** *17(14), 5210.* https://doi.org/doi:10.3390/ijerph17145210
49. Jones, R. 2020. How many medical beds does a country need? An international perspective. ***BJHCM*** 26(9), 248-259. https://doi.org/10.12968/bjhc.2020.0028
50. Jones, R. 2021. Were the hospital bed reductions proposed by English Clinical Commissioning Groups (CCGs) in the Sustainability and Transformation Plans (STPs) achievable? Insights from a new model to compare international bed numbers. ***Intl J Health Planning and Management*** 36(2), 459-481**.** https://doi.org/10.1002/hpm.3094
51. Jones, R. 2021. Multidisciplinary insights into health care financial risk and hospital surge capacity, Part 1: Nearness to death, infectious outbreaks, and Covid-19. ***Journal of Health Care Finance. 47(3),*** Vol. 47, No. 3, Winter 2021 (healthfinancejournal.com)
52. Jones, R. 2021. With 1.9 million hospital beds why is India struggling? ***Journal of Health Care Finance. 47(3),*** Special Guest Authors (healthfinancejournal.com)
53. Jones, R. 2021. Does the ageing population correctly predict the need for medical beds over the next 40 years? Part one: Fundamental principles ***BJHCM*** 27(8), ***doi: 10.12968/bjhc.2020.0156*** Does the ageing population correctly predict the need for medical beds?: Part one: fundamental principles | British Journal of Healthcare Management (magonlinelibrary.com)
54. Jones, R. 2021. Does the ageing population correctly predict the need for medical beds over the next 40 years? Part two: Wider implications. ***BJHCM*** 27(10), ***doi: 10.12968/bjhc.2021.0116*** Does the ageing population correctly predict the need for medical beds? Part two: wider implications | British Journal of Healthcare Management (magonlinelibrary.com)
55. Jones, R. 2021. A simple method to validate medical bed number calculations. ***Eur J Internal Medicine 94: P108-P109.*** https://doi.org/10.1016/j.ejim.2021.08.009
56. Jones, R. 2022. A pragmatic method to compare international critical care beds: implications to pandemic preparedness and non-pandemic planning. ***Int J Health Planning and Management 37(4), 2167-2182.*** https://doi.org/10.1002/hpm.3458
57. Jones, R. 2022. A model to compare international hospital bed numbers, including a case study on the role of indigenous people on acute 'occupied' bed demand in Australian states. Int J Environ Res Public Health 19: 11239. https://doi.org/10.3390/ijerph191811239

58. Jones, R. 2023. Addressing the knowledge deficit in hospital bed planning, defining an optimum region for the number of different types of hospital beds in an effective health care system. Addressing the Knowledge Deficit in Hospital Bed Planning, Defining An Optimum Region for the Number of Different Types of Hospital Beds in An Effective Health Care System[v1] | Preprints.org

**M. The Link Between Deaths (all-cause mortality) and Medical Emergency Admissions**

1. Jones, R. 2012. Diagnoses, deaths and infectious outbreaks. ***BJHCM*** 18(10), 539-548.
2. Jones, R. 2013. An unexplained increase in deaths during 2012. ***BJHCM*** 19(5), 248-253.
3. Jones, R. 2013. Analysing excess winter mortality: 2012/13. ***BJHCM*** 19(12), 601-605.
4. Jones, R. 2014. Increased deaths in 2012: which conditions? ***BJHCM*** 20(1), 45-47.
5. Jones, R. 2014. Trends in death and end-of-life costs in the UK. ***BJHCM*** 20(6), 298-299.
6. Jones, R. 2014. Trends in emergency admissions per death. ***BJHCM*** 20(9), 446-447.
7. Jones, R. 2015. A previously uncharacterized infectious-like event leading to spatial spread of deaths across England and Wales: Characteristics of the most recent event and a time series for past events. ***JAMMR*** 5(11), 1361-1380. doi: 10.9734/BJMMR/2015/14285
8. Jones, R. 2015. Unexplained infectious events leading to deaths and medical admissions in Belfast. ***BJHCM*** 21(1), 46-47.
9. Jones, R. 2015. Unexpected Increase in Deaths from Alzheimer's, Dementia and Other Neurological Disorders in England and Wales during 2012 and 2013. ***Journal of Neuroinfectious Diseases*** 6:172. doi: 10.4172/2314-7326.1000172
10. Jones, R. 2015. Influenza-like-illness, deaths and health care costs. ***BJHCM*** 21(12), 587-589.
11. Jones, R. 2016. The real reason for the huge NHS overspend? ***BJHCM*** 22(1), 40-42.
12. Jones, R. 2016. Rising emergency admissions in the UK and the elephant in the room. ***Epidemiology: Open Access*** 6(4), 1000261 doi: 10.4172/2161-1165.1000261
13. Jones, R. 2016. Deaths and the marginal changes in healthcare costs ***BJHCM*** 22(10), 503-509.
14. Jones, R. 2016. Trend in proportion of deaths occurring in hospital. ***BJHCM*** 22(11), 572-573.
15. Jones, R. 2017. In-hospital deaths, all-cause mortality and medical admissions. ***BJHCM*** 23(5), 239-240.
16. Jones, R. 2017. Anticipated NHS demand in 2017/18. ***J Paramed Pract*** 9(6), 236-237.
17. Jones, R. 2017. What government data on death rates fail to show. ***BJHCM*** 23(8), 572-573.
18. Jones, R. 2017. A reduction in acute thrombotic admissions during a period of unexplained increased deaths and medical admissions in the UK. ***European Journal of Internal Medicine*** 46: e31-e33. http://dx.doi.org/10.1016/j.ejim.2017.09.007
19. Jones, R. 2018. Volatility in emergency admissions per death. ***BJHCM*** 23(11), 554-556.
20. Jones, R. 2018. Admissions for certain conditions show explosive growth in England following a sudden and unexpected increase in deaths. ***European Journal of Internal Medicine***. 2018; 54: e33-e35. doi: https://doi.org/10.1016/j.ejim.2018.03.005
21. Jones, R. 2018. Do outbreaks of 'Disease X' regulate NHS beds and costs? ***BJHCM*** 24(4), 204-205.
22. Jones, R. 2018. Clinical workload trends. ***BJHCM*** 24(6), 308-309.
23. Jones, R. 2018. Deaths in the UK show a large increase in 2018. ***BJHCM*** 24(8), 410-411.
24. Jones, R. 2018. Will 2018 set a record for deaths? ***BJHCM*** 24(9), 464-465.
25. Jones, R. 2018. End-of-life, unusual syndromic symptoms, and periods of high physician workload. ***Achievements of Biology and Medicine*** 31(1), 46-51. *http://files.odmu.edu.ua/biomed/2018/01/d181_46.pdf*
26. Jones, R. 2019. A need for transparency and evidence-based discussion. ***J Paramed Pract*** 11(5), 219-220.
27. Jones, R. 2019. The calendar year fallacy: The danger of reliance on calendar year data in end-of-life capacity and financial planning. ***The International Journal of Health Planning and Management*** 3***34(4), e1533-e1543.*** https://doi.org/10.1002/hpm.2838
28. Jones, R. 2019. Austerity in the UK and poor health: were deaths affected? ***BJHCM*** 25(11), 337-347.

**N. Financial Risk in Healthcare <u>- see sections on deaths/admissions and small-area spatiotemporal patterns to understand the basis for the very high financial risk in healthcare. Financial and capacity risk are interlinked.</u>**

1. Jones, R. Financial and operational risk in healthcare. ***HMC Conference 'Re-shaping Acute Services for the 21st Century', Barbican Centre, London. 26*** September 2002. (99+) (PDF) Financial and operational risk in health care provision and commissioning | Rodney Jones - Academia.edu
2. Jones, R. 2004. Financial risk in healthcare provision and contracts. ***Proceedings of the 2004 Crystal Ball User Conference***, June 16-18th, 2004. Denver, Colarado, USA. (11) (PDF) Financial risk in healthcare provision and contracts (researchgate.net)
3. Jones, R. 2008. Financial risk in practice-based commissioning. ***BJHCM*** 14(5), 199-204.

4. Jones, R. 2008. Financial risk in health purchasing Risk pools. ***BJHCM*** 14(6), 240-245.
5. Jones, R. 2008. Financial risk at the PCT/PBC Interface. ***BJHCM*** 14(7), 288-293.
6. Jones, R. 2009. Emergency admissions and financial risk. ***BJHCM*** 15(7), 344-350.
7. Jones, R. 2009. The actuarial basis for financial risk in practice-based commissioning and implications to managing budgets. ***Primary Health Care Research & Development*** 10(3), 245-253. https://doi.org/10.1017/S1463423609990089
8. Jones, R. 2010. What is the financial risk in GP Commissioning? ***British Journal of General Practice*** 60(578), 700-701. https://www.ncbi.nlm.nih.gov/pmc/articles/PMC2930237/
9. Jones, R. 2010. Cyclic factors behind NHS deficits and surpluses. ***BJHCM*** 16(1), 48-50.
10. Jones, R. 2010. Do NHS cost pressures follow long-term patterns? ***BJHCM*** 16(4), 192-194.
11. Jones, R. 2010. Nature of health care costs and financial risk in commissioning. ***BJHCM*** 16(9), 424-430.
12. Jones, R. 2010. Trends in programme budget expenditure. ***BJHCM*** 16(11), 518-526.
13. Jones, R. 2011. Cycles in inpatient waiting time. ***BJHCM*** 17(2), 80-81.
14. Jones, R. 2012. Time to re-evaluate financial risk in GP commissioning. ***BJHCM*** 18(1), 39-48.
15. Jones, R. 2012. Gender ratio and cycles in population health costs. ***BJHCM*** 18(3), 164-165.
16. Jones, R. 2012. Why is the 'real world' financial risk in commissioning so high? ***BJHCM*** 18(4), 216-217.
17. Jones, R. 2012. Volatile inpatient costs and implications to CCG financial stability. ***BJHCM*** 18(5), 251-258.
18. Jones, R. 2012. Cancer care and volatility in commissioning. ***BJHCM*** 18(6), 315-324.
19. Jones, R. 2012. Gender and financial risk in commissioning. ***BJHCM*** 18(6), 336-337.
20. Jones, R. 2012. End of life care and volatility in costs. ***BJHCM*** 18(7), 374-381.
21. Jones, R. 2012. Age and financial risk in healthcare costs. ***BJHCM*** 18(7), 388-389.
22. Jones, R. 2012. High risk categories and risk pooling in healthcare costs. ***BJHCM*** 18(8), 430-435.
23. Jones, R. 2012. Year-to-year volatility in medical admissions. ***BJHCM*** 18(8), 448-449.
24. Jones, R. 2012. Risk in GP commissioning: the loss ratio. ***BJHCM*** 18(11), 605-606.
25. Jones, R. 2012. Financial risk in GP commissioning: lessons from Medicare. ***BJHCM*** 18(12), 656-657.
26. Jones, R. 2013. Financial risk and volatile elderly diagnoses. ***BJHCM*** 19(2), 94-96.
27. Jones, R. 2013. Financial risk and volatile childhood diagnoses. ***BJHCM*** 19(3), 148-149.
28. Jones, R. 2013. Environmental volatility and healthcare costs. ***BJHCM*** 19(4), 198-199.
29. Jones, R. 2013. What every GP needs to know about financial risk in commissioning. ***General Practice Online*** http://www.priory.com/family_medicine/GP_commissioning_risk.htm
30. Jones, R. 2013. The funding dilemma: a lagged cycle in cancer costs. ***BJHCM*** 19(12), 601-605.
31. Jones, R. 2014. Financial volatility in NHS contracts. ***BJHCM*** 20(10), 489-491.
32. Jones, R. 2016. The real reason for the huge NHS overspend? ***BJHCM*** 22(1), 40-42.
33. Jones, R. 2019. Financial risk in health and social care budgets. ***BJHCM*** 25(2), 79-84.
34. Jones, R. 2019. The calendar year fallacy: The danger of reliance on calendar year data in actuarial calculations. ***Intl J Health Plan Management 34(4), e1533-e1543.*** https://doi.org/10.1002/hpm.2838
35. Jones, R. 2021. Multidisciplinary insights into health care financial risk and hospital surge capacity, Part 1: Nearness to death, infectious outbreaks, and Covid-19. ***Journal of Health Care Finance. 47(3),*** Vol. 47, No. 3, Winter 2021 (healthfinancejournal.com)
36. Jones, R. 2021. Multidisciplinary insights into health care financial risk and hospital surge capacity, Part 2: High population density is associated with enhanced year-to-year volatility in many aspects of poor health including health care worker sickness absence. ***Journal of Health Care Finance. 47(3),*** Vol. 47, No. 3, Winter 2021 (healthfinancejournal.com)
37. Jones, R. 2021. Multidisciplinary insights into health care financial risk and hospital surge capacity, Part 3: Outbreaks of a new type or kind of disease create unique risk patterns and confounds traditional trend analysis. ***Journal of Health Care Finance. 47(3),*** Vol. 47, No. 3, Winter 2021 (healthfinancejournal.com)
38. Jones, R. 2021. Multidisciplinary insights into health care financial risk and hospital surge capacity, Part 4: What size does a health insurer or health authority need to be to minimise risk? ***Journal of Health Care Finance. 47(3),*** Vol. 47, No. 3, Winter 2021 (healthfinancejournal.com)
39. Jones, R. 2021. The epidemiology of health and social care cost and capacity shocks. ***Odessa Medical Journal*** 2021(5), doi: 10.54229/2226-2008-2021-5-9

**O. Limitations of the Healthcare Resource Group (HRG) Tariff** - ***This is intrinsically linked to financial risk (see above) and the affordability criteria for the business case in new hospitals***

1. Jones, R. 2008. Limitations of the HRG tariff: excess bed days. ***BJHCM*** 14(8), 354-355.
2. Jones, R. 2008. Limitations of the HRG tariff: day cases. ***BJHCM*** 14(9), 402-404.
3. Jones, R. 2008. A case of the emperor's new clothes? ***BJHCM*** 14(10), 460-461.
4. Jones, R. 2008. Limitations of the HRG tariff: the trim point. ***BJHCM*** 14(11), 510-513.
5. Jones, R. 2008. Costing orthopaedic interventions. ***BJHCM*** 14(12), 539-547.

6. Jones, R. 2009. Limitations of the HRG tariff: efficiency comparison. ***BJHCM*** 15(1), 40-43.
7. Jones, R. 2009. Limitations of the HRG tariff: the RCI. ***BJHCM*** 15(2), 92-95.
8. Jones, R. 2009. Limitations of the HRG tariff: local adjustments. ***BJHCM*** 15(3), 144-147.
9. Jones, R. 2010. A maximum price tariff. ***BJHCM*** 16 (3), 146-147.
10. Jones, R. 2010. Nature of health care costs and the HRG tariff. ***BJHCM*** 16(9), 451-452.
11. Jones, R. 2010. Emergency assessment tariff: lessons learned. ***BJHCM 16***(12), 574-583.
12. Jones, R. 2010. High efficiency or unfair financial gain? ***BJHCM*** 16(12), 585-586.
13. Jones, R. 2010. Is the HRG tariff fit for purpose? nhsManagers.net: Managers Briefing
14. Jones, R. 2011. Impact of the A&E targets in England. ***BJHCM*** 17(1), 16-22.
15. Jones, R. 2011. Costs of paediatric assessment. ***BJHCM*** 17(2), 57-63.
16. Jones, R. 2011. Is the short stay emergency tariff a valid currency? ***BJHCM*** 17(10), 496-497.
17. Jones, R. 2011. Limitations of the HRG tariff: the national average. ***BJHCM*** 17(11), 556-557.
18. Jones, R. 2011. Limitations of the HRG tariff: gross errors. ***BJHCM*** 17(12), 608-609.
19. Jones, R. 2012. Is the Health Resource Group (HRG) tariff fit for purpose? ***BJHCM*** 18(1), 52-53.
20. Jones, R. 2012. Limitations of the HRG tariff. Healthcare Analysis & Forecasting.
21. Jones, R. 2013. A guide to maternity costs - why smaller units cost more. ***British Journal of Midwifery*** 21(1), 54-59. https://www.magonlinelibrary.com/doi/abs/10.12968/bjom.2013.21.1.54

***P.*** **Funding & the Funding Formula - *this is intrinsically linked to financial risk (see above)***

1. Jones, R. 1994. GP Fundholding: Readies reckoner. ***Health Service Journal*** 104 (10th Feb), 31.
2. Jones, R. 2011. Infectious outbreaks and the capitation formula. ***BJHCM*** 17(1), 36-38.
3. Jones, R. 2013. A fundamental flaw in person-based funding. ***BJHCM*** 19(1), 32-38.
4. Jones, R. 2013. Population density and healthcare costs. ***BJHCM*** 19(1), 44-45.
5. Jones, R.; Kellet, J. 2018. The way healthcare is funded is wrong: it should be linked to deaths as well as age, gender and social deprivation. ***Acute Medicine*** 17(4), 189-193. See https://acutemedjournal.co.uk/original-articles/the-way-healthcare-is-funded-is-wrong-it-should-be-linked-to-deaths-as-well-as-age-gender-and-social-deprivation/
6. Jones, R. 2021. Multidisciplinary insights into health care financial risk and hospital surge capacity, Part 4: What size does a health insurer or health authority need to be to minimise risk? ***Journal of Health Care Finance. 47(3),*** Multidisciplinary insights into health care financial risk and hospital surge capacity, Part 4: What size does a health insurer or health authority need to be to minimise risk? | Jones, PhD | Journal of Health Care Finance (healthfinancejournal.com)

**Q. Outbreaks of a New Type of Infectious Immune Impairment Affecting Deaths and Medical Admissions - The World Health Organisation's Disease X?**

1. Jones, R. 2010. Unexpected, periodic and permanent increase in medical inpatient care: man-made or new disease. ***Medical Hypotheses*** 74: 978-83. http://dx. doi.org/10.1016/j.mehy.2010.01.011
2. Jones, R. 2010. Can time-related patterns in diagnosis for hospital admission help identify common root causes for disease expression. ***Medical Hypotheses*** 75: 148-154. http://dx. doi.org/10.1016/j.mehy.2010.02.009
3. Jones, R. 2010. The case for recurring outbreaks of a new type of infectious disease across all parts of the United Kingdom. ***Medical Hypotheses*** 75: 452-457. http://dx. doi.org/10.1016/j.mehy.2010.04.023
4. Jones, R. 2013. Do recuring outbreaks of a type of infectious immune impairment trigger cyclic changes in the gender ratio at birth? ***Biomedicine International*** 4(1), 26-39. https://www.bmijournal.org/index.php/bmi/article/download/27/25
5. Jones, R. 2013. Widespread outbreaks of a subtle condition leading to hospitalization and death. ***Epidemiology: Open access*** 4(3), 137. doi: 10.4172/2161-1165.1000137
6. Jones, R. 2014. Unexpected single-year-of-age changes in the elderly mortality rate in 2012 in England and Wales. ***JAMMR*** 4(16), 3196-3207. https://journaljammr.com/index.php/JAMMR/article/view/15127
7. Jones, R. 2015. Unexpected and Disruptive Changes in Admissions Associated with an Infectious-like Event Experienced at a Hospital in Berkshire, England around May of 2012. ***JAMMR*** 6(1), 56-76. Unexpected and Disruptive Changes in Admissions Associated with an Infectious-like Event Experienced at a Hospital in Berkshire, England around May of 2012 | ***JAMMR*** (journaljammr.com)
8. Jones, R. 2015. A previously uncharacterized infectious-like event leading to spatial spread of deaths across England and Wales: Characteristics of the most recent event and a time series for past events. ***JAMMR*** 5(11), 1361-1380. A Previously Uncharacterized Infectious-like Event Leading to Spatial Spread of Deaths Across England and Wales: Characteristics of the most Recent Event and a Time Series for Past Events | ***JAMMR*** (journaljammr.com)
9. Jones, R. 2015. Are emergency admissions contagious? ***BJHCM*** 21(5), 227-235.

10. Jones, R. 2015. Recurring outbreaks of an infection apparently targeting immune function, and consequent unprecedented growth in medical admission and costs in the United Kingdom: A review. ***Brit J Med and Medical Research*** 6(8), 735-770. Recurring Outbreaks of an Infection Apparently Targeting Immune Function, and Consequent Unprecedented Growth in Medical Admission and Costs in the United Kingdom: A Review | *JAMMR* (journaljammr.com)
11. Jones, R. 2016. A presumed infectious event in England and Wales during 2014 and 2015 leading to higher deaths in those with neurological and other disorders. ***J Neuroinfectious Diseases*** 7(1), 1000213 doi: 10.4172/2314-7326.1000213
12. Jones, R. 2017. A reduction in acute thrombotic admissions during a period of unexplained increased deaths and medical admissions in the UK. ***European Journal of Internal Medicine*** 46: e31-e33 doi: http://dx.doi.org/10.1016/j.ejim.2017.09.007
13. Jones, R. 2017. Age-specific and year of birth changes in hospital admissions during a period of unexplained higher deaths in England. ***European Journal of Internal Medicine*** 45: 2-4. doi: http://dx.doi.org/10.1016/j.ejim.2017.09.039
14. Jones, R. 2018. Periods of unexplained higher deaths and medical admissions have occurred previously – but were apparently ignored, misinterpreted, or not investigated. ***Eur J Internal Medicine*** 40: e18-e20. https://doi.org/10.1016/j.ejim.2017.11.004
15. Jones, R. 2018. Do outbreaks of 'Disease X' regulate NHS beds and costs? ***BJHCM*** 24(4), 204-205.
16. Jones, R. 2018. Deaths in 2017 reached a new (unexpected) high. ***BJHCM*** 24(5), 256-257.
17. Jones, R. 2019. Unusual international behaviour of deaths suggests a possible new kind of disease outbreak. Healthcare Analysis & Forecasting. Via SSRN http://ssrn.com/abstract=3364795
18. Jones, R. 2019. Why are deaths in the UK behaving in such a peculiar way? Healthcare Analysis & Forecasting. doi: 10.13140/RG.2.2.13685.42728

**R. Small-Area Spatiotemporal Patterns in the Spread of a New Type or Kind of Infectious Disease (The WHO Disease X?)**

1. Jones, R. 2013. A recurring series of infectious-like events leading to excess deaths, emergency department attendances and medical admissions in Scotland. ***Biomedicine International*** 4(2), 72-86. http://www.bmijournal.org/index.php/bmi/article/view/35
2. Jones, R. 2014. Infectious-like Spread of an Agent Leading to Increased Medical Admissions and Deaths in Wigan (England), during 2011 and 2012. ***JAMMR*** 4(28), 4723-4741 Infectious-like Spread of an Agent Leading to Increased Medical Admissions and Deaths in Wigan (England), during 2011 and 2012 | ***JAMMR*** (journaljammr.com)
3. Jones, R.; Beauchant, S. 2015. Spread of a new type of infectious condition across Berkshire in England between June 2011 and March 2013: Effect on medical emergency admissions. ***JAMMR*** 6(1), 126-148. Small Area Spread of a New Type of Infectious Condition across Berkshire in England between June 2011 and March 2013: Effect on Medical Emergency Admissions | *JAMMR* (journaljammr.com)
4. Jones, R. 2015. Simulated rectangular wave infectious-like events replicate the diversity of time-profiles observed in real-world running 12-month totals of admissions or deaths. ***FGNAMB*** 1(3), 78-79. doi: 10.15761/FGNAMB.1000114
5. Jones, R. 2015. A new type of infectious outbreak? ***SMU Medical Journal*** 2(1), 19-25. http://smu.edu.in/content/dam/manipal/smu/documents/Journal%20Issue%203/A%20New%20Type%20of%20Infectious%20Outbreak.pdf
6. Jones, R. 2015. Small area spread and step-like changes in emergency medical admissions in response to an apparently new type of infectious event. ***FGNAMB 1***(2), 42-54. doi: 10.15761/FGNAMB.1000110
7. Jones, R. 2015. Infectious-like spread of an agent leading to increased medical hospital admission in the North East Essex area of the East of England. ***FGNAMB*** 1(3), 98-111. doi: 10.15761/FGNAMB.1000117
8. Jones, R. 2015. A time series of infectious-like events in Australia between 2000 and 2013 leading to extended periods of increased deaths (all-cause mortality) with possible links to increased hospital medical admissions. ***International Journal of Epidemiologic Research*** 2(2), 53-67. http://ijer.skums.ac.ir/article_12869_2023.html
9. Jones, R. 2015. Deaths and international health care expenditure. ***BJHCM*** 21(10), 491-493.
10. Jones, R. 2016. A fatal flaw in mortality-based disease surveillance. ***BJHCM*** 22(3), 143-145.
11. Jones, R. 2016. Deaths in English Lower Super Output Areas (LSOA) show patterns of very large shifts indicative of a novel recurring infectious event. ***SMU Medical Journal*** 3(2), 23-36. https://pdfs.semanticscholar.org/c3aa/71a1b78e053cba4a871093dd43aa896d9ef6.pdf
12. Jones, R. 2016. A regular series of unexpected and large increases in total deaths (all-cause mortality) for male and female residents of mid super output areas (MSOA) in England and Wales: How high-level analysis can miss the contribution from complex small-area spatial spread of a presumed infectious agent. ***Fractal Geometry and Nonlinear Analysis in Medicine and Biology*** 2(2), 1-13. doi: 10.15761/FGNAMB.1000129

13. Jones, R. 2017. Outbreaks of a presumed infectious agent associated with changes in fertility, stillbirth, congenital abnormalities and the gender ratio at birth. ***JAMMR*** 20(8), 1-36. doi: 10.9734/BJMMR/2017/32372

**14.** Jones, R. 2017. Outbreaks of a presumed infectious pathogen creating on/off switching in deaths. ***SDRP Journal of Infectious Diseases Treatment and Therapy*** 1(1), 1-6. http://www.openaccessjournals.siftdesk.org/articles/pdf/Outbreaks-of-a-presumed-infectious-pathogen-creating-on-off-switching-in-deaths20170606102727.pdf

*15.* Jones, R. 2017. Year-to-year variation in deaths in English Output Areas (OA), and the interaction between a presumed infectious agent and influenza in 2015. ***SMU Medical Journal* 4(2),** 37-69. http://smu.edu.in/content/dam/manipal/smu/smims/Volume4No2July2017/SMU%20Med%20J%20(July%202017.%20-%204.pdf

16. Jones, R. 2017. Deaths and medical admissions – what is happening in the UK? ***FGNAMB*** 3(1), 1. doi: 10.15761/FGNAMB.1000143

17. Jones, R. 2018. Deaths and medical admissions in the UK show an unexplained and sustained peak after 2011. ***Eur J Internal Med 47: e14-e16.*** DOI: http://dx.doi.org/10.1016/j.ejim.2017.09.021

**S. Cytomegalovirus (CMV) and Human Disease**

1. Jones, R. 2011. CMV and health care costs. ***BJHCM*** 17(4), 168-169.
2. Jones, R. 2013. Could cytomegalovirus be causing widespread outbreaks of chronic poor health? In ***Hypotheses in Clinical Medicine,*** pp 37-79, Eds M. Shoja, et al. New York: Nova Science Publishers Inc.
3. Jones, R. 2014. A Study of an unexplained and large increase in respiratory deaths in England and Wales: Is the pattern of diagnoses consistent with the potential involvement of Cytomegalovirus? ***JAMMR*** 4(33), 5179-5192. A Study of an Unexplained and Large Increase in Respiratory Deaths in England and Wales: Is the Pattern of Diagnoses Consistent with the Potential Involvement of Cytomegalovirus? | ***JAMMR*** (journaljammr.com)
4. Jones, R.; Goldeck, D. 2014. Unexpected and unexplained increase in death due to neurological disorders in 2012 in England and Wales: Is cytomegalovirus implicated? ***Medical Hypotheses*** 83(1), 25-31. http://dx.doi.org/10.1016/j.mehy.2014.04.016
5. Jones, R. 2015. Roles for cytomegalovirus in infection, inflammation and autoimmunity. In ***Infection and Autoimmunity***, 2nd Edition, Eds: N Rose, et al. Elsevier: Amsterdam. Chapter 18, p 319-357. doi:10.1016/B978-0-444-63269-2.00068-4
6. Jones, R. 2015. An unexpected increase in adult appendicitis in England 2000/01 to 2012/13), Could cytomegalovirus (CMV) be a risk factor? ***JAMMR*** 5(5), 579-603. An Unexpected Increase in Adult Appendicitis in England 2000/01 to 2012/13), Could Cytomegalovirus (CMV) be A Risk Factor? | ***JAMMR*** (journaljammr.com)
7. Jones, R. 2016. Is cytomegalovirus involved in recurring periods of higher than expected death and medical admissions, occurring as clustered outbreaks in the northern and southern hemispheres? ***JAMMR*** 11(2), 1-31. https://journaljammr.com/index.php/JAMMR/article/view/9833
8. Jones, R. 2017. International outbreaks of a novel type of infectious immune impairment: A call to action. **Досягнення біології та медицини** (***Achievements of Biology and Medicine - transl)*** 29(1), 75-81. http://files.odmu.edu.ua/biomed/2017/01/d171_75.pdf or BIO171a.pm6 (hcaf.biz)
9. Jones, R. 2017. Outbreaks of a presumed infectious agent associated with changes in fertility, stillbirth, congenital abnormalities and the gender ratio at birth. ***JAMMR*** 20(8), 1-36. Outbreaks of a Presumed Infectious Agent Associated with Changes in Fertility, Stillbirth, Congenital Abnormalities and the Gender Ratio at Birth | ***JAMMR*** (journaljammr.com)

Dr Rodney Jones has over 30-years' experience in health care demand forecasting, capacity and bed number planning, and financial risk in health care purchasing and commissioning. He has published over 300 papers and reports on these topics. His 'Research Interest' score places him in the top 5%of international researchers out of 25+ million ResearchGate members (top 25% for researchers of the same age). His papers have over 1,000 citations https://www.researchgate.net/profile/Rodney_Jones. Dr Rodney Jones can be contacted at: hcaf_rod@yahoo.co.uk or +44 (0)7890 640399 **ORCHID ID: 0000-0002-4810-7638**

**Supplementary Material S2.** Age-based forecasting underestimates future admissions for diseases of the appendix.

Diseases of the appendix (ICD-10 primary diagnoses K35 to K38) have been chosen to illustrate the flaws in age-based forecasting because the diagnosis (based on all information available at discharge) is likely to be highly accurate. The bulk of admissions in this group are for appendicitis.

Figure S2.1 shows the actual and predicted trends for various age groups for hospital admissions in England for the 22-year period 1998/99 to 2019/20. The trend for each age group has been calculated based on age-banded admissions in 1998/99 extrapolated forward using population estimates in each age band. Population estimates are rebased at each census, hence in 2000 and 2010. The 2020 rebased estimates are not yet available.

Data regarding hospital admissions in England is from NHS Digital [3], while population data for England is from the Office for National Statistics [4].

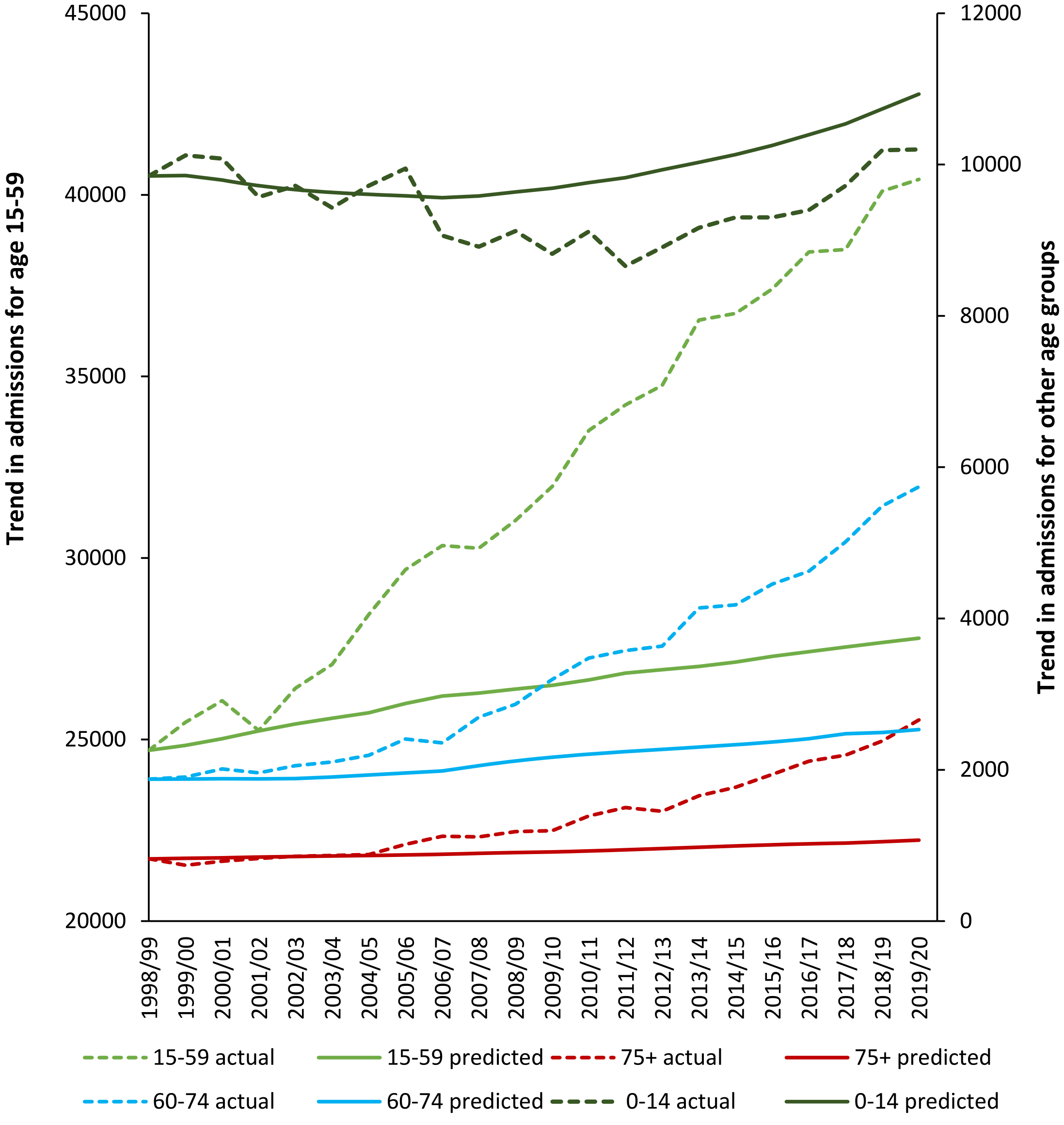


**Figure S2.1.** Actual and predicted trends in England for diseases of the appendix using four age bands over the period 1998/99 to 2019/12.

The agreement between the actual and predicted trends is somewhat variable, namely, up to 2001/02 for age 15–59, roughly up to 2003/04 for age 60–74, up to 2005/06 for age 75+ and up to 2005/06 for age 0–14. Beyond 2005/06 the actual admissions for age 0–14 lie below the age-based forecast and by 2019/20 are 7% lower than the forecast. By 2019/20 admissions in the 75+ age band had increased by 149% compared to the age-based forecast while the total admissions across all ages was 40% higher than the age-based forecast.

Note that the proportion of patients aged 15–59 was 66.3% in 1998/99, rising to a maximum of 71.2% in 2011/12 and 2012/13, and decreasing to 68.2% by 2019/20.

In conclusion, age-based forecasting only worked for a maximum of seven years into the future for the youngest age band and for about five years for age 75+.

Given the emphasis of this study on the unexpected effects of 3000 known species of human pathogens on human health it is worth noting that appendicitis is now increasingly considered to have an infectious aetiology with numerous parasites and other pathogens acting to trigger the disease [5-9].

Within a context of pathogen interference, the incidence of complicated appendicitis increased during the COVID-19 pandemic [10].

For a more detailed discussion specific to appendicitis, see publication [S.6] in the Supplement S1.

$

**Supplementary material S3.** Use of the new method to investigate trends in occupied beds in England

Of necessity the analysis of bed numbers in Sections 4 and 7 of the main text are restricted to the reported number of available beds. To remedy this deficiency this section will explore the trends in ***occupied*** beds in England for several specialties using the new method. Occupied beds includes both emergency and elective care and includes an assumed 8 hour stay for all same day (zero day) stay patients.

In England a patient is admitted to a particular specialty which is usually under the care of a consultant surgeon or physician who specializes in that aspect of care. A consultant can have multiple specialties. The medical specialties show increasing sub-specialization over time and the occupied beds in earlier years, up to around 2004/05 will be low since care in some hospitals will have been delivered by a general physician rather than one with a sub-specialty.

The data starts in 1998/99 which is at the far-right hand side of the Figures. The crude mortality rate reaches a minimum of 8.5 in 2011 and 8.6 in 2014. It is now steadily rising. A line of constant beds per 1000 deaths is shown on each chart. Data on occupied beds in England is from NHS Digital [3], while crude mortality is from the Office for National Statistics [41].

Note that these are occupied beds and allowance must be made at local level for the size of the hospital with an implied bed occupancy margin and the level of local deprivation (as age standardized mortality rate). Both are discussed in relevant sections in the main text.

In all cases further international research is required on the topic of occupied as opposed to available beds.

Figure S3.1 commences with the trends for palliative care, infectious diseases, and nuclear medicine (far right side). Note the step up for infectious diseases in 2003/04 as the care of infectious diseases expanded. Hence possible optimum somewhere above an intercept = 3.

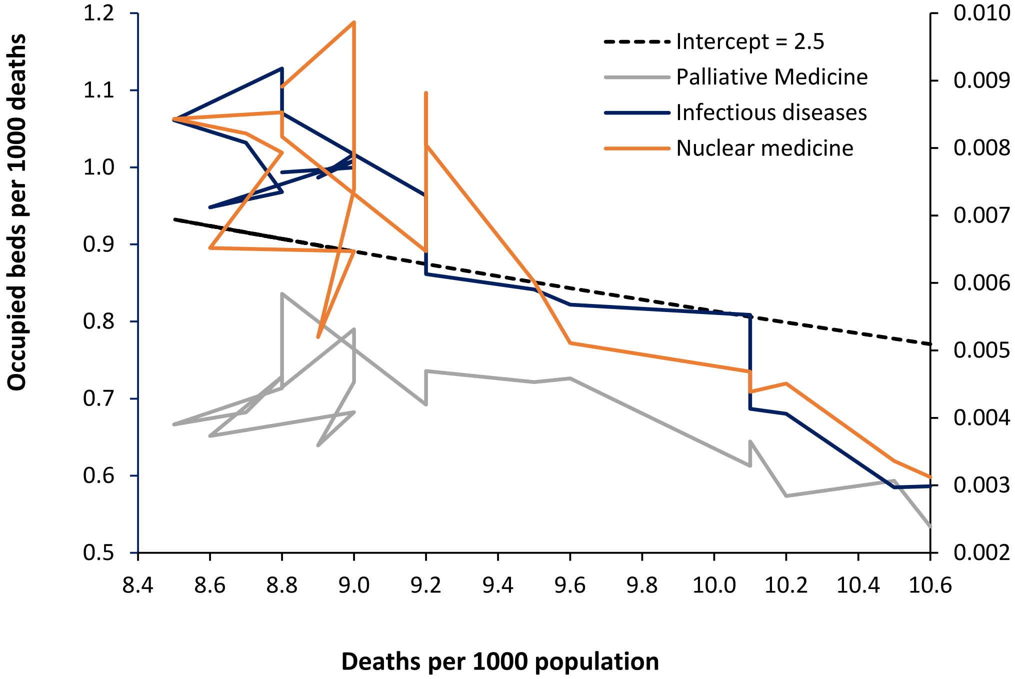


**Figure S3.1.** Occupied beds per 1000 deaths in England for the specialties palliative medicine, infectious diseases, and nuclear medicine.

Palliative care bed provision in the UK is not straightforward since significant amounts of such care is delivered by charitably funded hospices and the occupied bed days do not get reported since they are not conducted in an NHS hospital. In 2017 it was estimated that there were around 2175 occupied hospice beds in the UK (how many hospice beds in england - Search (bing.com)). Hence optimum for palliative care is somewhere more than an intercept = 2.5.

Nuclear medicine involves the application of radioactive substances in the diagnosis and treatment of diseases. Much of this care will be conducted as an outpatient radiology study. Hence somewhere above 0.01 occupied beds per death probably for the treatment aspect of the specialty.

Figure S3.2 shows the trends for rehabilitation and haematology. For rehabilitation some care appears to have been transferred to private units, hence the optimum is probably around an intercept = 15. The optimum for haematology is probably slightly above an intercept =15.

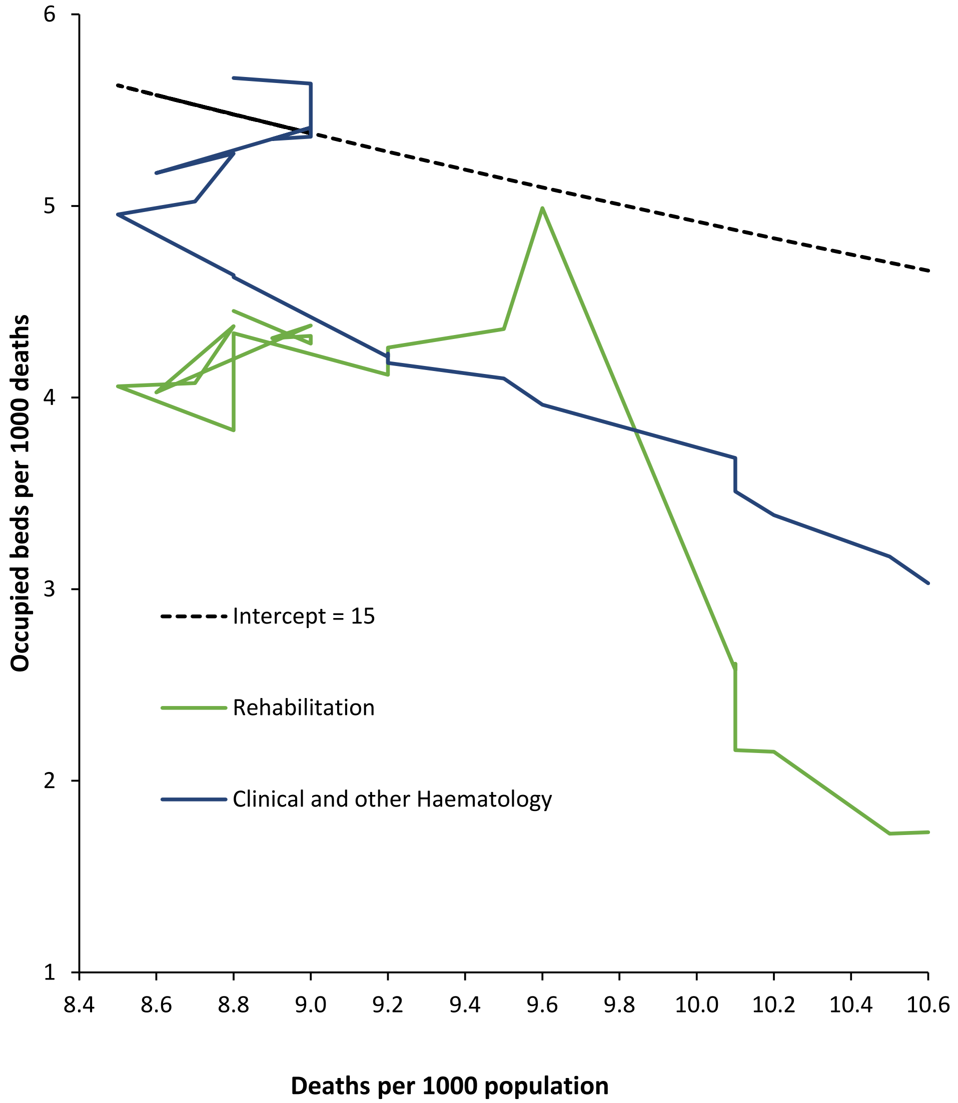


**Figure S3.2.** Occupied beds per 1000 deaths in England for the specialties of rehabilitation and haematology.

Figure S3.3 shows the trends for geriatric medicine (left hand axis), gastroenterology and gynaecology (both right hand axis).

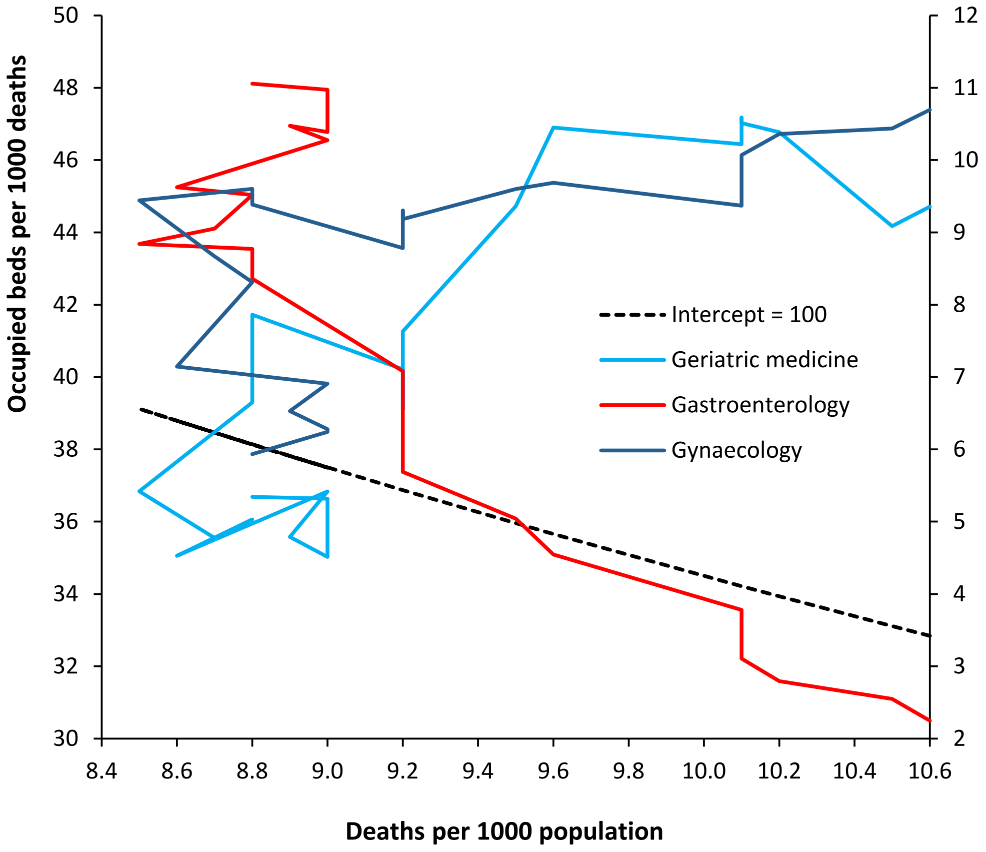


**Figure S3.23.** Occupied beds per 1000 deaths in England for the specialties of geriatric medicine, gastroenterology, and gynaecology.

Gynaecology initially shows a slight decline as more elective surgery is performed as a day case. The decline in gynaecology occupied beds after 2011/12 is a direct result on the austerity measures implemented after the financial crash. On this occasion elective care will simply accumulate on the waiting list. The optimum is perhaps somewhere around an intercept = 30.

The decline in geriatric medicine after 2005/06 will partly reflect the introduction of the specialty 'acute internal medicine' which will operate in medical assessment units. Some of the bed days will therefore occur as acute internal medicine, and care will then be passed to a geriatric medicine specialist.

Gastroenterology shows continuous growth as this specialty expands its base of specialist consultants. Optimum provision is probably somewhere above an intercept = 30.

These examples have demonstrated how the new method can be applied at specialty level as long as relevant history is accounted for.

**Supplementary material S4.** The age standardized mortality rate (ASMR), and higher bed demand due to social deprivation

Figure S4.1 shows the relationship between deaths per 1000 population and ASMR for world countries and UK local authorities. As is to be expected there is a slight upward trend as deaths per 1000 population increases, however, the key feature is that at the same value of deaths per 1000 population there is a very wide range in ASMR values. Hence the two are effectively independent variables. R-squared for the UK is only 0.157, while for world countries it is only 0.0024.

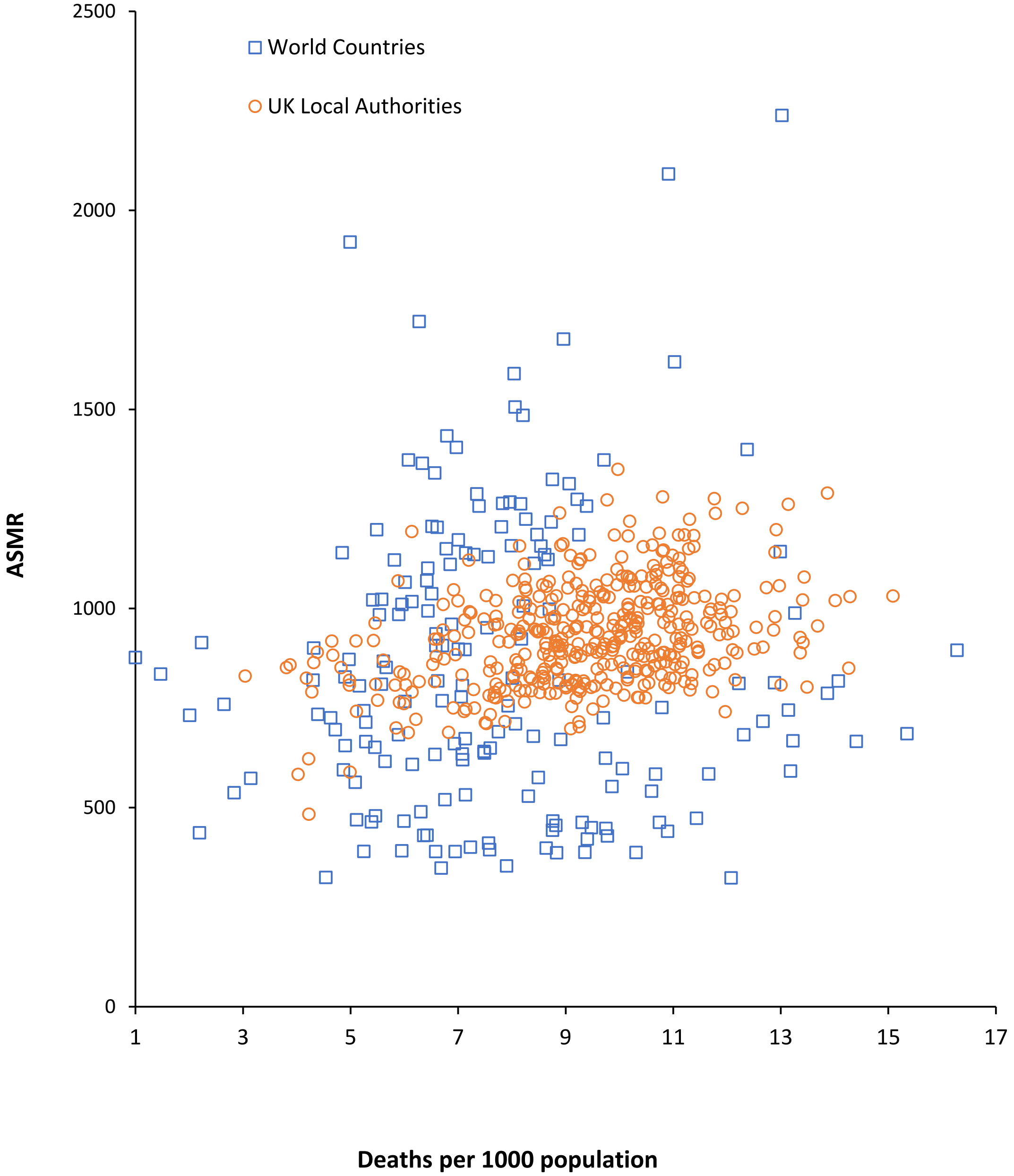


**Figure S4.1**. Relationship between deaths per 1000 population and age standardized mortality rate (ASMR) for UK local authorities and world countries. Data from [43,44].

Table S4.1 gives the ASMR values for the 50 countries with the lowest ASMR [43]. The values for Sweden, England and the UK are highlighted.

**Table S4.1.** The 50 countries having the lowest ASMR in 2019 [44], deaths per 1000 population (DpT) and the ratio of ASAMR relative to Japan.

| Country | DpT | ASMR | R Ratio to Japan |
|---|---|---|---|
| Japan | 12.1 | 323.3 | 1.00 |
| Singapore | 4.5 | 324.1 | 1.00 |
| Iceland | 6.7 | 348.0 | 1.08 |
| Switzerland | 7.9 | 353.2 | 1.09 |
| Spain | 8.8 | 385.9 | 1.19 |
| Italy | 10.3 | 386.8 | 1.20 |
| France | 9.4 | 387.5 | 1.20 |
| Luxembourg | 6.9 | 389.2 | 1.20 |
| Australia | 6.6 | 389.2 | 1.20 |
| Israel | 5.2 | 389.8 | 1.21 |
| South Korea | 6.0 | 391.1 | 1.21 |
| Norway | 7.6 | 394.0 | 1.22 |
| **Sweden** | **8.6** | **397.9** | **1.23** |
| Malta | 7.2 | 400.4 | 1.24 |
| Canada | 7.6 | 410.3 | 1.27 |
| Austria | 9.4 | 420.8 | 1.30 |
| Finland | 9.8 | 428.4 | 1.33 |
| New Zealand | 6.4 | 429.5 | 1.33 |
| Ireland | 6.4 | 430.6 | 1.33 |
| Kuwait | 2.2 | 436.4 | 1.35 |
| Portugal | 10.9 | 439.9 | 1.36 |
| Netherlands | 8.8 | 443.1 | 1.37 |
| Slovenia | 9.8 | 447.4 | 1.38 |
| Belgium | 9.5 | 449.5 | 1.39 |
| England | 8.8 | 455.0 | 1.41 |
| Denmark | 9.3 | 462.4 | 1.43 |
| Germany | 10.7 | 462.5 | 1.43 |
| Colombia | 5.4 | 463.8 | 1.43 |
| Peru | 6.0 | 465.5 | 1.44 |
| United Kingdom | 8.8 | 465.9 | 1.44 |
| Panama | 5.1 | 469.1 | 1.45 |
| Greece | 11.4 | 473.0 | 1.46 |
| Costa Rica | 5.5 | 479.2 | 1.48 |
| Chile | 6.3 | 489.0 | 1.51 |
| Cyprus | 6.8 | 519.4 | 1.61 |
| United States | 8.3 | 528.2 | 1.63 |
| Thailand | 7.1 | 531.6 | 1.64 |
| Maldives | 2.8 | 537.0 | 1.66 |
| Czechia | 10.6 | 541.0 | 1.67 |
| Cuba | 9.9 | 552.8 | 1.71 |
| Turkey | 5.1 | 563.6 | 1.74 |
| Jordan | 3.1 | 573.2 | 1.77 |
| Albania | 8.5 | 575.2 | 1.78 |
| Poland | 10.7 | 583.8 | 1.81 |
| Estonia | 11.7 | 584.3 | 1.81 |
| Croatia | 13.2 | 591.3 | 1.83 |
| Iran | 4.9 | 594.4 | 1.84 |
| Uruguay | 10.1 | 597.5 | 1.85 |
| Tunisia | 6.1 | 608.0 | 1.88 |
| Paraguay | 5.6 | 615.7 | 1.90 |

To test if ASMR is relevant measure for higher bed demand due to relative deprivation we first need to check if it is well correlated with other measures for deprivation which have been shown to measure hospital admissions or bed demand.

Many countries have developed measures for social deprivation at small area level which have been used to indicate the strength of the relationship between deprivation and utilization of the emergency department and hospital admissions [45].

The UK has developed two measures for social deprivation. The first is the index of multiple deprivation (IMD) which can be applied at small area and local authority level [46]. A study from Scotland shows that emergency admissions have a far greater dependence on IMD than elective admissions, and that this is more so for males than females [47]. This is also the case for emergency department and outpatient attendances. In the USA 30-day mortality after surgery increases with neighbourhood deprivation [48].

The known relationship between IMD and healthcare utilization can be used to check if ASMR is a valid measure of bed demand. This is illustrated In Figure S3.2. where a nonlinear relationship between the two can be seen. Hence, by extrapolation ASMR is an equally valid measure of the overall effects of social deprivation. The great advantage is that it is available for international comparison.

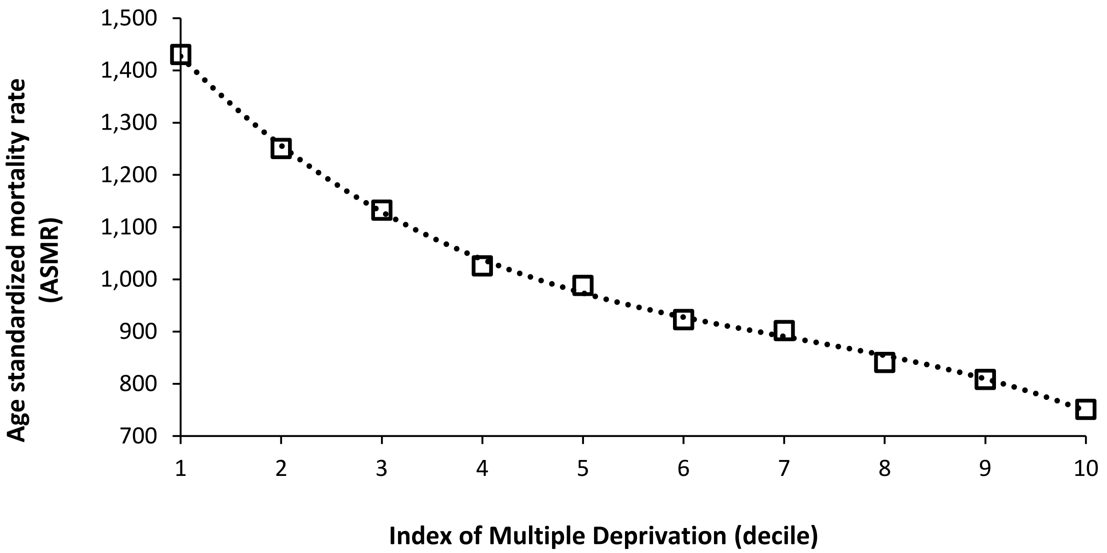


**Figure S4.2.** Direct link between ASMR and IMD demonstrated. All data is aggregated at the same small area level over which IMD is calculated [49]. Data was standardized to the 2013 European population.

For the second of the two measures for deprivation the UK has developed a small-area system of social groups called the Output Area Classification (OAC). The OAC uses the methods employed to construct consumer groups and applies this to data collected during the 10-yearly Census to derive a detailed set of social groups [50]. While such social groups provide profound insight into patterns of health care utilization [45,B.10-11,C.14] they are specific to the UK.

The health behaviours arising from social groups will however be ultimately expressed in the ASMR for each location. Once again ASMR is considered an appropriate basis to calculate the higher need for hospital beds based on relative need. It is assumed that the slope of the relationship will be applied differentially between elective and emergency demand and between specialties. This assumption relies on a degree of local and national analysis.

It should be noted that ASMR shows volatility over time simply because deaths show high volatility, and this is demonstrated in Figure S3.3. Observe that 2019 was a historically low ASMR year, hence the need to establish baseline trends [G.6]. Hence ASMR is also reflecting the short-term factors affecting human health.

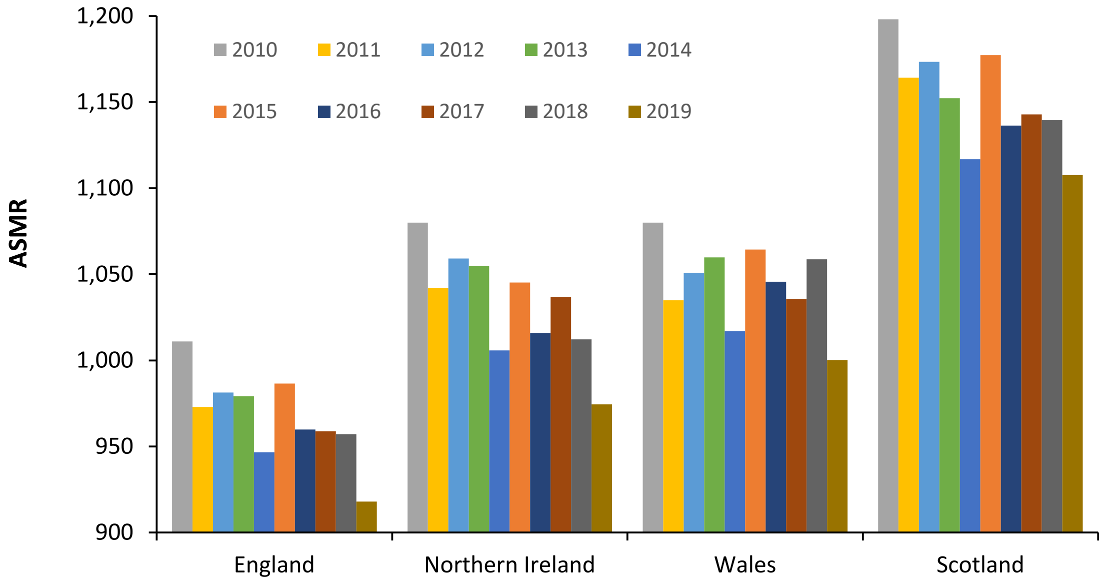


**Figure S4.3**. As with all healthcare utilization ASMR also shows considerable volatility over time. Data is from the ONS [49].

Further to the issue of volatility Figure S4.4 shows the trend in ASMR for a selection of world countries while S4.5 shows the linked volatility in both ASMR and the crude mortality rate for the UK. Especially note how volatility in Figure S4.4 appears to reduce as the size of the country increases. Any form of healthcare planning based on the average will give misleading results.

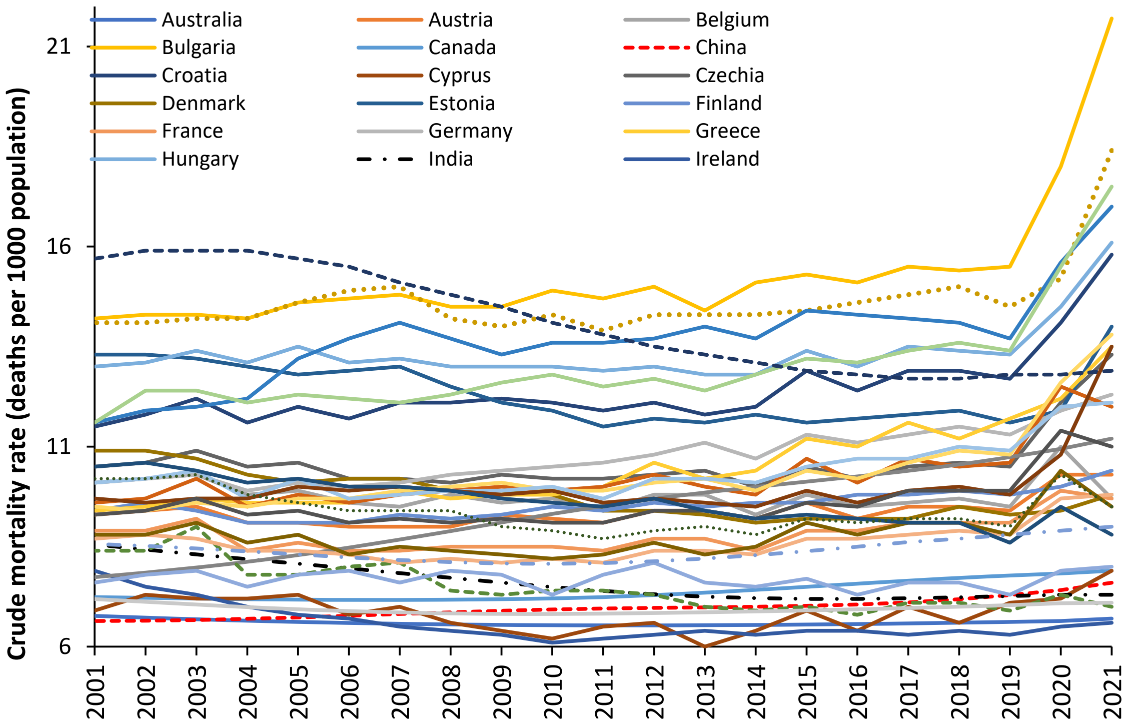


**Figure S4.4**. Volatility in the trend in ASMR for a selection of world countries [44].

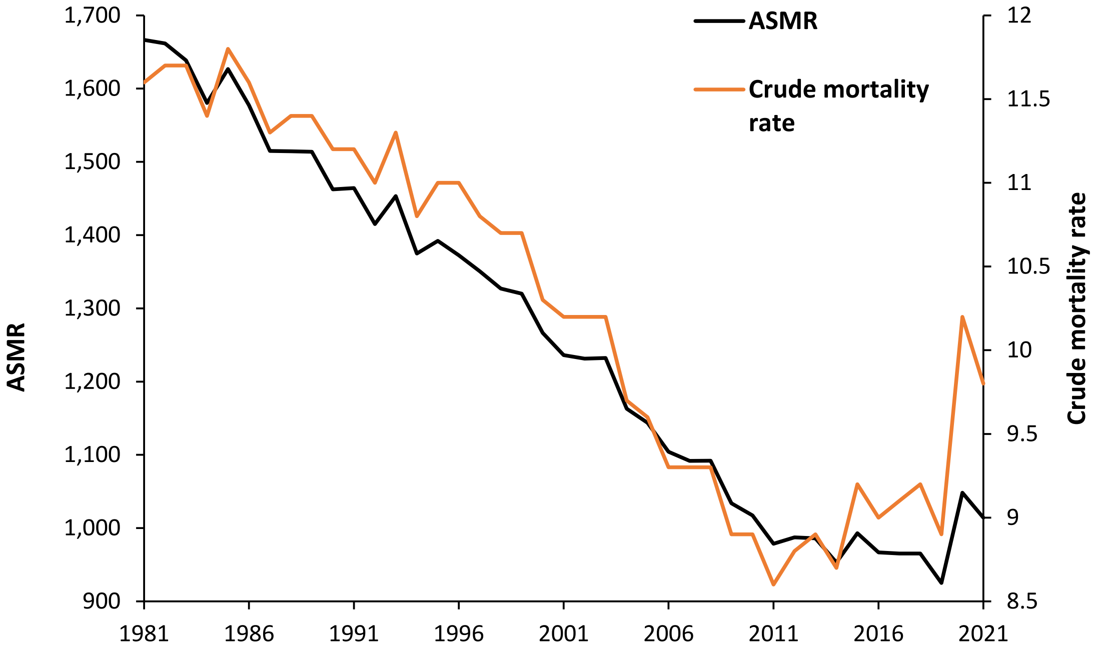


**Figure S4.5**. Volatility in the trend in ASMR and crude mortality rate for the UK [44]. From [Vital statistics in the UK: births, deaths and marriages - Office for National Statistics (ons.gov.uk)]

The profoundly important issues regarding volatility are addressed in Section 11 of the main text.

**Supplementary material S5.** The slope of the relationship between ASMR and hospital bed numbers

We need to address the issue regarding the slope of the relationship between the age standardized mortality rate (ASMR) and the number of hospital beds per 1000 deaths. To estimate the relationship between beds per 1000 deaths and ASMR the data in Figure 1 for the wealthy countries in the medium, high, and very high bed number categories the value of beds per 1000 deaths for each country was first adjusted to an equivalent number assuming the same deaths per 1000 population as for the UK. This was then plotted against the ASMR for these countries. Given that ASMR was available as an additional variable, some countries were moved between the medium/high/very high categories to give the resulting trends shown in Figure S5.1. Note that the slope of the relationship varies between a 12 to 14 unit increase in beds per 1000 deaths for every 100 unit increase in ASMR. This relationship only holds for relatively wealthy countries with an ASMR up to 1100. The estimate of the slope using international bed numbers in Figure S5.1 relied on the reallocation of countries between three groups of nominally medium, high, and very high categories. This allocation was subjective and could be an underestimate.

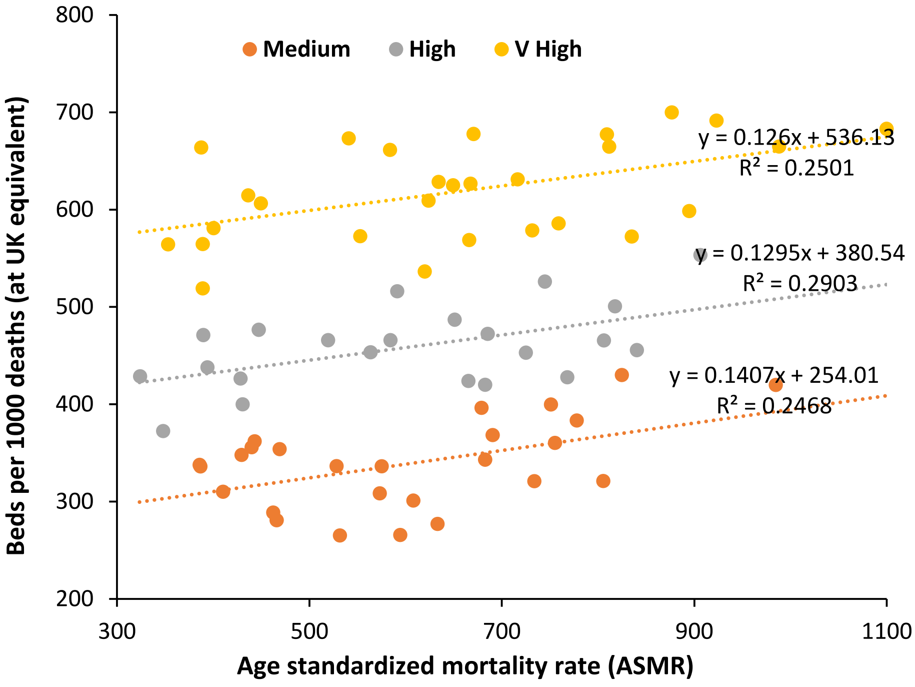


**Figure S5.1.** Relationship between beds per 1000 deaths (at crude mortality rate equal to the UK) and the age standardized mortality rate (ASMR).

To further validate this relationship occupied acute beds per 1000 deaths data for US states was plotted against state ASMR. Data from [L.48,L.50]. The resulting trend gave a slope of 15.6 units of occupied beds per 1000 deaths for every 100 unit increase in ASMR which is very close to the estimate of 12 to 14 derived above. However, the R-squared was very low.

Finally, this estimate can be triangulated using data from the UK [49,50]. Three estimates can be derived namely 0.28 (28 per 100 units of ASMR) using data from all four countries in the UK, 0.34 (34 per 100 units of ASMR) using data from England and Scotland, and 1.01 (101 per 100 units of ASMR) using data from England, Northern Ireland, and Wales.

A potential confounding factor is that the magnitude of the apparent slope between UK countries is strongly influenced by the Barnet formula which allocates block funds to the devolved governments of Scotland, Northern Ireland, and Wales to establish 'equal' funding relative to England [52]. The devolved governments are free to apply the block funds to education, healthcare, etc., however they choose. In 2018/19 the three countries received more block funds per capita of +36% Wales, +48% Scotland and +54% Northern Ireland all relative to England. In the same year England had around 247

occupied beds per 1000 deaths compared with around 312 in Scotland and Northern Ireland (at equivalent deaths per 1000 population) and 328 in Wales [L.49]. The impact of such allocation is made more complex by the way in which each devolved government chooses to run the NHS.

According to the Barnet formula bed utilization in Scotland should lie between Northern Ireland and Wales. Clearly this is not the case in terms of occupied beds per 1000 deaths, hence, the three different estimates for the apparent slope. The upper apparent slope of 1.01 is considered too high leaving two estimates at 0.28 and 0.34 compared to 0.12 to 0.14 estimated earlier. The real value probably lies somewhere between the two extremes.

Please note that this estimate only applies to the total of all bed types. The slope of the relationship is likely to be different depending on the specialty of care or the condition, i.e., appendicitis versus heart failure, etc. Elective versus emergency care will also have a different value of the slope.

Table A5.1 then uses the above relationships to evaluate the discrepancy between occupied acute beds per 1000 deaths in US states expected from their ASMR (relative to the US average) and the actual difference. In this Table the data is first corrected for the differing levels of deaths per 1000 population in each state. This correction is applied along lines of equivalent bed provision such that all states are at the average deaths per 1000 population for the USA. The calculation is then performed based on the value of the slope of total beds versus ASMR from Supplementary materials S5.

**Table A5.1.** Expected change in beds per 1000 deaths in US states due to ASMR and the actual difference. All relative to the US average for ASMR. Data from [53].

| State | Deaths per 1000 population | ASMR | Effect relative to US average beds per 1000 deaths: Slope = 0.13 | Effect relative to US average beds per 1000 deaths: Slope = 0.34 | Actual difference in bed supply (beds per 1000 deaths) |
|---|---|---|---|---|---|
| Wyoming | 818 | 784 | 1 | 3 | -140 |
| Idaho | 765 | 751 | -3 | -8 | -85 |
| Utah | 564 | 727 | -6 | -16 | -75 |
| Vermont | 877 | 730 | -6 | -15 | -56 |
| Montana | 929 | 778 | 0 | 1 | -53 |
| Oregon | 866 | 748 | -4 | -9 | -48 |
| Colorado | 656 | 705 | -9 | -24 | -45 |
| Washington | 746 | 717 | -8 | -20 | -43 |
| Iowa | 950 | 748 | -4 | -9 | -41 |
| New Mexico | 825 | 778 | 0 | 1 | -40 |
| Louisiana | 949 | 937 | 21 | 55 | 55 |
| Missouri | 975 | 851 | 10 | 26 | 57 |
| West Virginia | 1200 | 963 | 24 | 64 | 58 |
| Tennessee | 987 | 921 | 19 | 50 | 59 |
| Kentucky | 1011 | 941 | 22 | 56 | 61 |
| Alabama | 1050 | 959 | 24 | 62 | 63 |
| Massachusetts | 850 | 715 | -8 | -21 | 90 |
| Florida | 964 | 714 | -8 | -21 | 90 |
| Arizona | 790 | 721 | -7 | -18 | 92 |
| New York | 802 | 701 | -10 | -25 | 121 |

As can be seen there are gross discrepancies ranging from 140 fewer occupied beds per 1000 deaths in Wyoming through to 121 more in New York. These reflect the known disparities in healthcare between US states [54,55]. Such gross discrepancies arise from barriers to access due to long distances to the nearest hospital in some states, through to the proportion of the population with health insurance and/or who can afford the copayments, etc. In the more affluent states, there will be supply induced demand.

One study showed that in the USA an estimated 1 in 10 residents experienced low access to any hospital with emergency surgical capabilities, and 1 in 4 experienced low access to hospitals with advanced clinical resources. Communities with high proportions of uninsured, publicly insured, and racial and ethnic minority groups in micropolitan and rural regions were at the greatest risk of being in low access areas [56]

**Supplementary material S6.** Issues regarding length of stay (LOS)

The ‘apparent’ LOS for hospitals is often miscalculated by including large numbers of zero day stay admissions [K.1-9]. The real overnight stay LOS is calculated by dividing bed days by admissions after excluding all types of same day stay admissions.

This is illustrated in Figure S6.1 which shows the trend in the real overnight LOS for several specialty groups in England. Data is from NHS Digital [3].

If the trend to day surgery is very strong, then the trend for the real overnight LOS is likely to increase because the more complex patients/procedures remain in the overnight stay pool. This is evident for Ear, Nose and Throat (ENT).

Note that the other specialties have reached an asymptote in recent years and may even be trending upward due to the residual complex patient effect and due to the increasing pool of elderly patients.

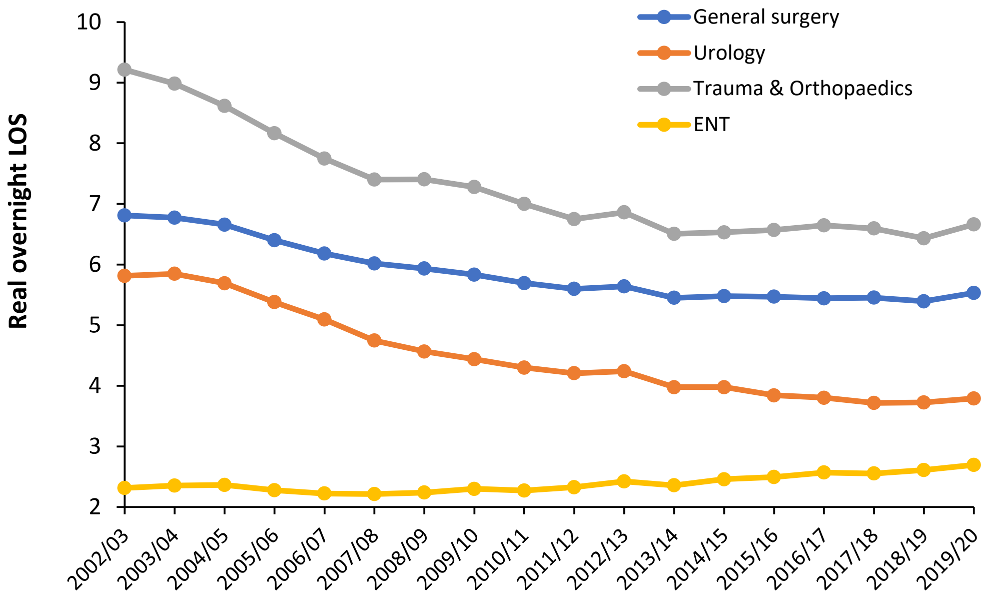


**Figure S6.1.** Trend in the real overnight stay length of stay (LOS) in England

Dare I suggest that this is one area where LOS can be manipulated. The bed provision for all types of zero or same day stay admissions should be calculated separately based on real-time LOS data.

**Supplementary Material S7.** Measures of volatility in deaths and volatility in bed demand

Figure S7.1 shows various measures for the year-to-year volatility (as a 12-month moving total of deaths) for local government areas across the UK. Data on monthly deaths in England and Wales is from the Office for National Statistics [95].

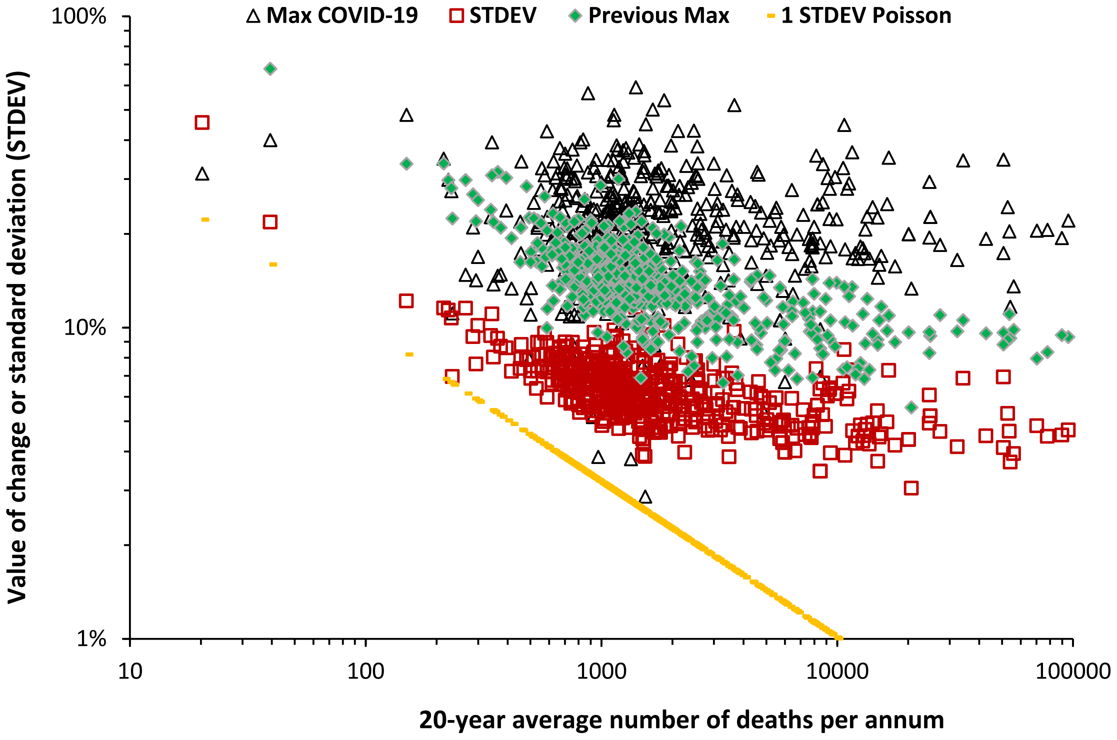


**Figure S7.1**. Various measures of the volatility associated with a 12-month moving total of deaths for local authority areas, regions, and countries in the UK.

In Figure S7.1 maximum change during the COVID-19 pandemic and for the maximum change prior to the arrival of COVID-19 is calculated as the maximum percentage difference between 12-month periods, i.e., January to December 2020 versus January to December 2021. Move forward by one month and recalculate. Locate the point for the maximum difference. The data series commences for the total of monthly deaths January to December 2000. One standard deviation (STDEV) of Poisson variation is calculated as the square root of the average number of deaths divided by the average number of deaths. The STDEV for the time series is calculated using the usual method for calculating STDEV based upon the time series for percentage differences.

As can be seen in Figure S7.1 the year-to-year volatility increases as size reduces. Below 100 deaths Poisson-based variation begins to dominate, although not completely.

As the spatial unit (number of deaths per annum) increases the volatility reaches an asymptote because the highs/lows in the smaller areas begin to cancel out. Such spatial granularity is a key feature of infectious outbreaks. Note the very high spatial granularity for the COVID-19 outbreaks and the minority of locations where COVID-19 had very little impact on deaths.

Translate this variation into volatile admissions at local hospital level to get an indication of the surge capacity required for different sized hospitals in different locations. Each location will be characterized by unique networks for social contact plus the lived population density.

Figure S7.2 investigates the year-to-year volatility in specialty-level NHS bed demand during the pre-COVID-19 era.

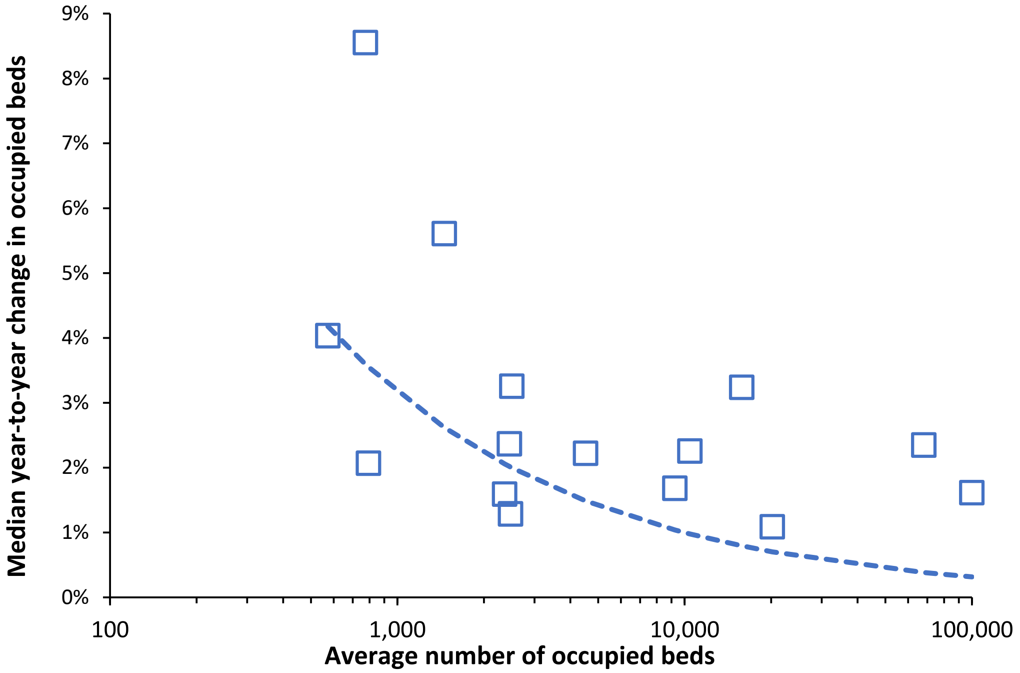


**Figure S7.2**. Median year-to-year volatility in occupied beds in England, measured at specialty level [19].

Median value for the year-to-year volatility in the number of occupied beds in England for various specialty groups (1999/00 to 2018/19). The dashed line gives an approximation for the contribution from Poisson randomness. The value shown is the square root of the number of occupied beds divided by the number of occupied beds.

As would be expected the volatility decreases with size, however, note that some specialty groups show far higher environmental sensitivity than others. In this respect the medical group has the highest year-to-year volatility relative to the line for Poisson randomness. Insufficient medical beds will effectively destabilize a hospital.

When these figures are translated from national to local level the total size of most hospitals is below 1,000 beds and so the minimum volatility at total hospital level will be well above 5% and at specialty level will be above 10%. The need for an appropriate average bed occupancy margin should be obvious. The often quoted figure of 85% is nothing other than an often repeated myth or fairy tale [L.1-58].

**Supplementary material S8.** Patterns in sickness absence

Figure S8.1 shows the moving 12-month average sickness absence rate for NHS staff. Data sources [E.1-6]. The shape of the trends is not consistent with winter influenza outbreaks. Note that the Omicron variant seemingly substitutes sickness absence for mortality.

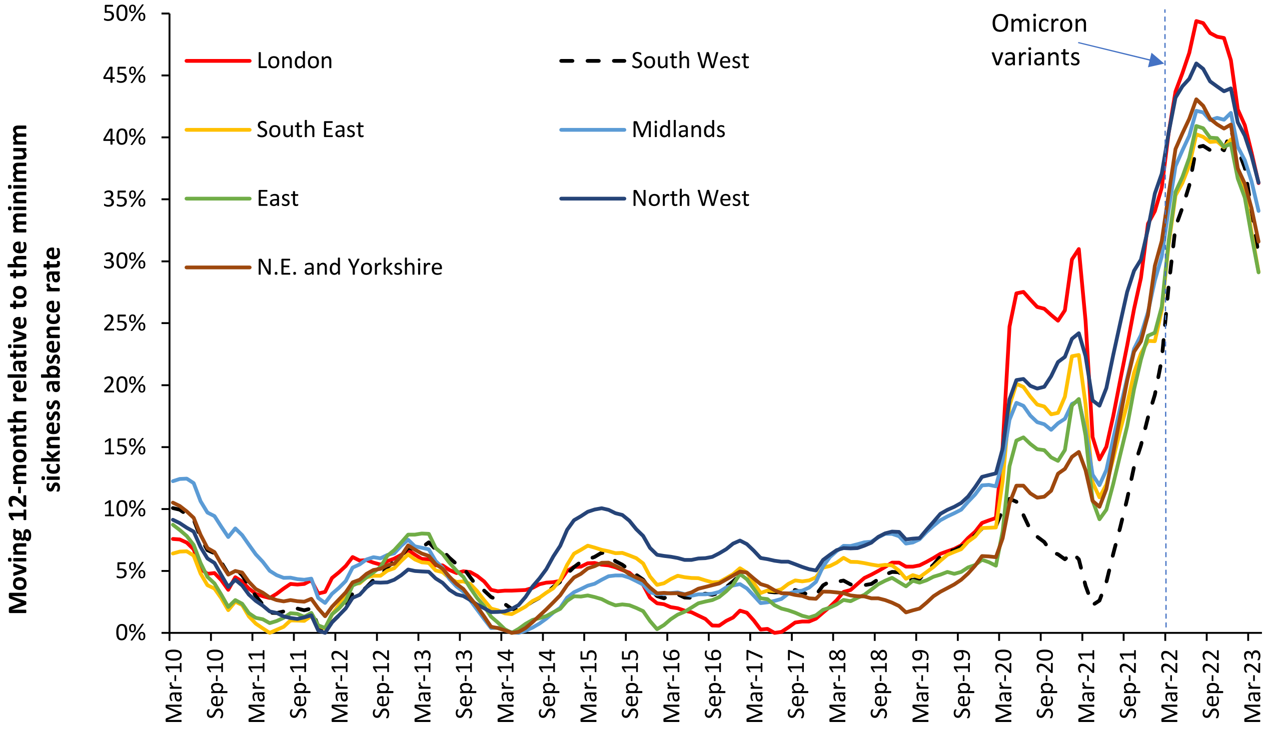

**Supplementary material S9.** Using long-term trends to inform bed planning

Figure S9.1 commences with the long-term trends in occupied beds in England as occupied beds per death or per birth for obstetrics and paediatrics. This analysis uses high level specialty groups. All trends are relative to the median value in each series. For all groups the total occupied beds include an assumed 8 hour stay for any type of same day (zero day stay) admission. This includes both day case surgery and any other type of same day stay admission. Actual counts of same day admissions are available from 2013/14 onward and trends have been extrapolated backward. Same day stay does not make a large contribution to the total and so any uncertainty in the backward extrapolation makes a minimal impact.

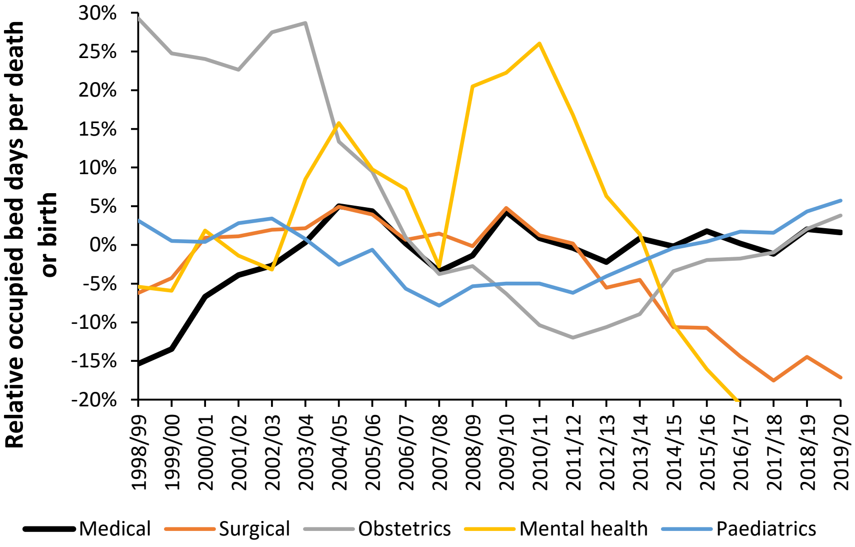


**Figure S9.1**. Trend in occupied bed days per death or births for various specialty groups in England [].

There are several key observations. Firstly, note the exaggerated peaks in mental health/psychiatric bed occupancy which can only be rationally explained by pathogen outbreaks [M.1-29,Q.1-18,R.1-17]. The surgical and medical groups also show such peaks. Psychiatric bed demand declines after 2010/11 as care in the community is introduced. Undulations in demand still occur despite the downward trend.

The trough in obstetric group bed days per death was the outcome of insufficient beds due to rising births. Births reached a minimum in 2001/02 and then rose to a 23% higher maximum in 2011/12. The minimum in 2001/02 lured hospitals into incorrectly closing obstetric group beds. As births begin to decline after 2011/12 so the occupied beds per birth begins to rise back toward what may be considered safe levels of length of stay [K.9]. Such undulations in births arise out from the World War II baby boom and are somewhat predictable [L.20]. However, there is never any central guidance on the issue. The NHS is left to blunder around. Paediatrics follows a somewhat similar trend since paediatric bed demand likewise depends on births with highest demand in the first year of life.

The trend downward for surgical bed demand after 2010/11 is partly due to a greater emphasis on day surgery and partly due to a rapidly expanding inpatient waiting list. The actual bed demand is simply hidden in a queue. In addition, bed availability also declines implying that overnight surgery is curtailed.

The rise in medical group bed demands up to 2004/05 is most likely due to increasing levels of intervention especially in the field of cardiovascular medicine. As for psychiatric bed demand, medical bed demand likewise shows a series of (inexplicable) peaks and troughs. Once again refer to the extensive series of papers relating to infectious outbreaks and human health.

Figure S9.2 addresses the issue regarding the proportion of surgical group admissions which are an emergency.

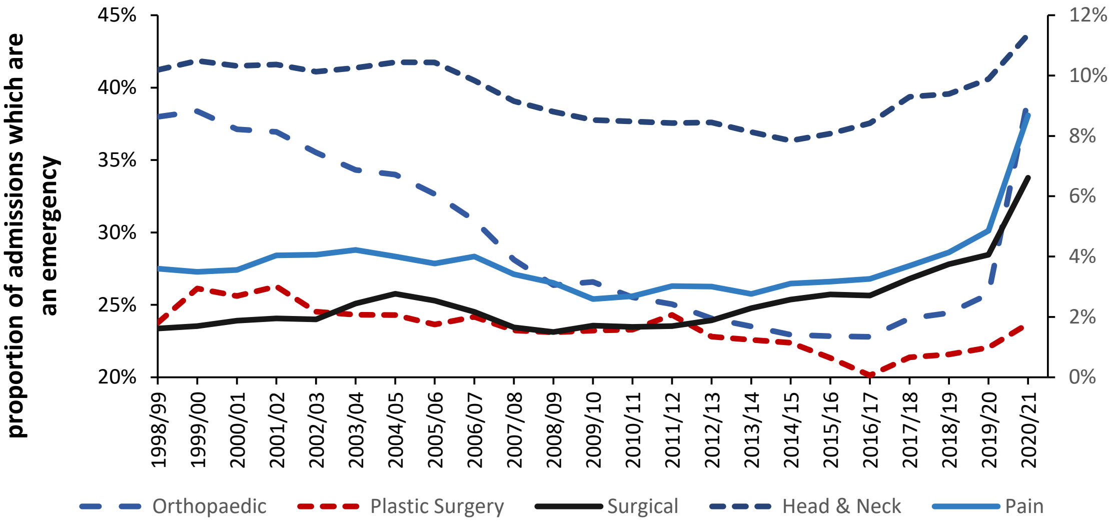


**Figure S9.2**. Trend in proportion of surgical group admissions which are an emergency.

Each type/specialty shows its own unique trends. A trend upward is usually instigated when bed supply becomes so constrained that elective activity is diverted into a waiting list. Note the large shift up in the first year of the COVID-19 pandemic when routine elective surgery is effectively halted. This then skews the proportion toward emergency admissions. Bed availability has a huge impact on the fundamental functioning of all specialties.

This section has not been exhaustive but is meant to illustrate that that application of simplistic bed models into a complex system is guaranteed to fail.
